# Reconstitution of T Cell Proliferation under Arginine Limitation: Activated Human T Cells Take Up Citrulline *via* L-Type Amino Acid Transporter 1 and Use It to Regenerate Arginine after Induction of Argininosuccinate Synthase Expression

**DOI:** 10.3389/fimmu.2017.00864

**Published:** 2017-07-24

**Authors:** Anke Werner, Miriam Koschke, Nadine Leuchtner, Claudia Luckner-Minden, Alice Habermeier, Johanna Rupp, Christin Heinrich, Roland Conradi, Ellen I. Closs, Markus Munder

**Affiliations:** ^1^Third Department of Medicine (Hematology, Oncology, and Pneumology), University Medical Center of the Johannes Gutenberg University Mainz, Mainz, Germany; ^2^Department of Pharmacology, University Medical Center of the Johannes Gutenberg University Mainz, Mainz, Germany; ^3^Transfusion Center, University Medical Center of the Johannes Gutenberg University Mainz, Mainz, Germany; ^4^Research Center for Immunotherapy, University Medical Center of the Johannes Gutenberg University Mainz, Mainz, Germany

**Keywords:** T lymphocyte, arginine, citrulline, T cell metabolism, amino acid transporter

## Abstract

In the tumor microenvironment, arginine is metabolized by arginase-expressing myeloid cells. This arginine depletion profoundly inhibits T cell functions and is crucially involved in tumor-induced immunosuppression. Reconstitution of adaptive immune functions in the context of arginase-mediated tumor immune escape is a promising therapeutic strategy to boost the immunological antitumor response. Arginine can be recycled in certain mammalian tissues from citrulline *via* argininosuccinate (ASA) in a two-step enzymatic process involving the enzymes argininosuccinate synthase (ASS) and argininosuccinate lyase (ASL). Here, we demonstrate that anti-CD3/anti-CD28-activated human primary CD4^+^ and CD8^+^ T cells upregulate ASS expression in response to low extracellular arginine concentrations, while ASL is expressed constitutively. ASS expression peaked under moderate arginine restriction (20 µM), but no relevant induction was detectable in the complete absence of extracellular arginine. The upregulated ASS correlated with a reconstitution of T cell proliferation upon supplementation of citrulline, while the suppressed production of IFN-γ was refractory to citrulline substitution. In contrast, ASA reconstituted proliferation and cytokine synthesis even in the complete absence of arginine. By direct quantification of intracellular metabolites we show that activated primary human T cells import citrulline but only metabolize it further to ASA and arginine when ASS is expressed in the context of low amounts of extracellular arginine. We then clarify that citrulline transport is largely mediated by the L-type amino acid transporter 1 (LAT1), induced upon human T cell activation. Upon siRNA-mediated knockdown of LAT1, activated T cells lost the ability to import citrulline. These data underline the potential of citrulline substitution as a promising pharmacological way to treat immunosuppression in settings of arginine deprivation.

## Introduction

T lymphocyte activation relies on reprogramming of key metabolic pathways and the sufficient availability of nutrients like glucose and amino acids (1, 2). Deficiency of the amino acid arginine is crucially involved in inflammation- and cancer-associated immunosuppression with profound impairment of T cell functions (3–6). In the inflammatory microenvironment, arginine is metabolized to ornithine and urea by the enzyme arginase, expressed by different types of myeloid cells, such as alternatively activated macrophages, myeloid-derived suppressor cells (MDSC) (7), or conventional granulocytes (8). Arginase is localized intracellularly and/or is liberated into the extracellular space. In an arginine-deprived milieu, T cell proliferation is abolished (9) by inhibition of cell cycle progression in G_0_–G_1_ phase (10), while T cell production of cytokines is either also impaired (e.g., IFN-γ) or largely unaffected (e.g., IL-2) (11, 12). As a consequence, inhibition of arginase-expressing MDSC (7) or supplementation of arginine to T cells (6) leads to increased efficiency of antitumoral T cells.

Cells can acquire arginine *via* uptake of the amino acid through specialized transmembrane transport proteins (13, 14) or by intracellular recycling from autophagic protein degradation (5, 15). We have recently shown that activated human T cells dramatically increase arginine import, due to the specific upregulation of cationic amino acid transporter-1 (CAT-1) (16), and that this induction of CAT-1 is necessary for efficient T cell proliferation (16). Human T cells also respond to arginine deprivation with the induction of autophagy, likely as a means to gain access to arginine intracellularly. This cytoprotective mechanism preserves T cell viability (17), but can of course not sustain cell proliferation. An alternative rescue strategy for cells to cope with limited amounts of extracellular arginine is to metabolize the non-proteinogenic amino acid citrulline *via* the enzymes argininosuccinate synthase (ASS) and argininosuccinate lyase (ASL) into arginine. ASS catalyzes the ATP-dependent condensation of citrulline and aspartate to argininosuccinate (ASA), which is then further metabolized *via* ASL to arginine and fumarate. These enzymatic reactions are part of the hepatic urea cycle, but ASS and ASL are coexpressed in a variety of cell types, in which they coordinately synthesize arginine (18). In humans, a large part of plasma citrulline is synthesized in the intestine from glutamine through the glutamate–ornithine pathway. The kidneys take up most of intestinally released citrulline, convert it to arginine *via* ASS and ASL and this contributes about 60% of whole body *de novo* arginine synthesis (19). In the immune system, the importance of the citrulline–arginine pathway has already been demonstrated for macrophages that can both take up citrulline (20) and derive it from their nitric oxide synthase-mediated enzymatic degradation of arginine into NO and citrulline (21). Citrulline can then be used for endogenous arginine synthesis *via* ASS and ASL for further NO generation (citrulline–NO cycle).

Can citrulline also serve as a rescue substrate for human T cells in the context of limiting amounts of arginine? Human T-ALL Jurkat cells use this strategy successfully: arginine depletion suppresses cell proliferation, but leads to an increase in citrulline uptake and expression of ASS. Consequently, Jurkat cell proliferation is completely rescued upon citrulline supplementation (22). These findings in the leukemic human T cell line have been recapitulated in murine primary T cells where the citrulline-induced rescue of proliferation is completely dependent on T cell ASS expression (23, 24). In contrast, in the absence of arginine, proliferation of human peripheral blood mononuclear cells (PBMCs) (23) or purified T cells (16) cannot be rescued by supplementation of citrulline. These results are in agreement with older literature, demonstrating that resting human normal T lymphocytes do not express ASS (25, 26). It therefore remained unclear if and how human primary T cells (i) take up citrulline, (ii) express ASS and ASL, and (iii) use citrulline for reconstitution of their suppressed functions under arginine depletion. Defining the parameters of a potential citrulline-mediated T cell functional rescue in the setting of restricted arginine availability would potentially be of therapeutic relevance, especially for tumor therapy. Systemic application of citrulline is possible, since the amino acid has a high bioavailability leading to significant increases in concentrations in the blood stream with no signs of toxicity (27, 28).

## Materials and Methods

### Reagents

Unless otherwise specified, reagents were purchased from Sigma Aldrich (Steinheim, Germany) or Roth (Karlsruhe, Germany). All amino acids were used as l-isomers.

### T Cell Purification and Cultivation

This study was carried out in accordance with the recommendations of the Rhineland Palatine Medical Association Ethics Committee with written informed consent from all subjects. All subjects gave written informed consent in accordance with the Declaration of Helsinki. The protocol was approved by the Rhineland Palatine Medical Association Ethics Committee. PBMCs were separated from blood of healthy donors as described before (16). CD3^+^ T cells were isolated by negative selection using T cell enrichment Kit (Stemcell Technologies, Cologne, Germany). CD4^+^ and CD8^+^ T cells were isolated from PBMCs by positive selection (Miltenyi Biotec, Bergisch Gladbach, Germany). T cell purity was tested by flow cytometry and was reproducibly more than 96%. Prior to the start of the experiments, T cells were cultivated in RPMI 1640, supplemented with 10% FBS (PAA), 2 mM glutamine, 100 U/mL penicillin, and 0.1 mg/mL streptomycin.

### T Cell Activation

T lymphocytes were activated with anti-CD3/anti-CD28-coupled Dynabeads^®^ (ratio 5:1, Life Technologies, Darmstadt, Germany) at a total number of 1 × 10^6^ cells in 200 µL RPMI 1640 medium without glutamine, lysine, and arginine (Sigma Aldrich), supplemented with (final concentrations) 10% dialyzed FBS (Sigma Aldrich), 2 mM glutamine, 200 µM lysine, 100 U/mL penicillin, 0.1 mg/mL streptomycin, and arginine at the indicated concentration, respectively.

### Proliferation Assay and IFN-γ ELISA

T cell proliferation was assessed after the indicated incubation times by the incorporation of [^3^H]-thymidine (5 μCi/mL, specific activity: 6.7 Ci/mmol, Amersham Biosciences, Freiburg, Germany) over 16 h. Before scintillation counting, cells were washed three times with PBS, lysed using MicroScint 20 (Perkin Elmer, Waltham, MA, USA), and analyzed with a Tri-Carb^®^2810 TR (Perkin Elmer, Waltham, MA, USA). Alternatively, T cells were labeled with 25 µM 5-(and 6)-carboxyfluorescein diacetate succinimidyl ester (CFSE, Invitrogen, Carlsbad, CA, USA) for 15 min according to the manufacturer’s instructions. After 96 h of activation, cells were analyzed by flow cytometry (FL-1). For IFN-γ detection, T cell culture supernatants were harvested at the indicated time points and IFN-γ was determined using the BD OptEIA Human IFN-γ ELISA Set according to the manufacturer’s instructions (Becton Dickinson, Heidelberg, Germany).

### Western Blot

5 × 10^6^ T cells were lysed on ice for 30 min in 200 µL RIPA buffer (Sigma Aldrich), supplemented with protease inhibitor cocktail (Complete mini, Roche, Mannheim, Germany) or in 50 mM Tris, pH 7.5, containing 1% Brij^®^ O10, 1 mM phenylmethylsulfonyl fluoride, 10 mM NaF, 1 mM Na_3_VO_4_, 1 µg/mL leupeptin, and 1.5 µg/mL pepstatin. Cell debris was removed by centrifugation at 4°C. 30 µg protein were separated by sodium dodecyl sulfate-polyacrylamide gel electrophoresis and transferred on Amersham Protran nitrocellulose or Amersham Hybond polyvinylidene fluoride membrane (GE Healthcare, Little Chalfont, UK) by wet or semi-dry blotting. After blocking with 5% non-fat dry milk in TBST buffer (10 mM Tris, 150 mM NaCl, and 0.05% Tween 20) for 2 h, the membranes were incubated with antibodies against human LAT1 (Cell Signaling Technology, #5347, 1:5,000), CD98 (4F2hc) (Santa Cruz, sc-9160, 1:1,000), ASS (Sigma Aldrich, HPA020896, 1:1,000), ASL (Sigma Aldrich, HPA016646, 1:1,000), and overnight at 4°C. As reference for equal protein loading, antibodies against glyceraldehyde 3-phosphate dehydrogenase (Cell Signaling, #2118, 1:10,000) or ERK 1 (Santa Cruz, K-23, 1:1,000) were used. After incubation with horseradish peroxidase-conjugated anti-rabbit IgG (Santa Cruz Biotechnology, Dallas, TX, USA or Calbiochem) in 5% non-fat dry milk in TBST buffer for 1 h at room temperature, antibody reactivity was assessed by adding Western Lightning^®^ Plus ECL (Perkin Elmer) and exposing the membranes to Amersham Hyperfilm ™ ECL (GE Healthcare).

### Citrulline Uptake in Human T Lymphocytes

T cells, which were left unstimulated or stimulated for 24 h in the presence of different arginine concentrations, were washed twice with 1× Locke’s solution (154 mM NaCl, 5.6 mM KCl, 1 mM MgCl_2_, 10 mM HEPES, 3.6 mM NaHCO_3_, 2 mM CaCl_2_, and 5.6 mM glucose), supplemented with 2% bovine serum albumin (BSA). For uptake measurements in the absence of sodium, NaCl in 1× Locke’s solution was replaced by choline chloride. Cells were then incubated in the presence of 1 μCi/mL l-[ureido-^14^C]citrulline (specific activity: 55 mCi/mmol, Hartmann Analytic, Braunschweig, Germany) at 37°C for different time periods, washed three times in ice cold 1× Locke’s solution plus BSA and lysed with 2% SDS. [^14^C]-labeled citrulline taken up by the cells was then quantified by scintillation counting in 2 mL Irga-Safe Plus with the Tri-Carb^®^ 2810 TR (Perkin Elmer, Waltham, MA, USA).

### Uptake Studies in LAT1/4F2hc-Expressing *Xenopus laevis* Oocytes

Expression of human LAT1 and the associated glycoprotein 4F2hc in *X. laevis* oocytes was achieved *via* injection of 20 ng LAT1 and 10 ng 4F2hc complementary DNA-derived RNA (cRNA) as previously described (29). After 48 h of transporter expression, oocytes were washed three times in ice-cold uptake solution (100 mM NaCl, 2 mM KCl, 1 mM MgCl_2_, 1 mM CaCl_2_, 5 mM HEPES, and 5 mM Tris, pH 7.5), supplemented with 20 µM citrulline and then incubated in the same solution additionally containing 1 μCi/mL l-[ureido-^14^C]citrulline (specific activity: 55 mCi/mmol, Hartmann Analytic) at 21°C. After 15 min, oocytes were washed 5× with ice-cold uptake solution, lyzed individually in 2% SDS, and radioactivity was quantified in 2 mL Irga-Safe Plus with the Tri-Carb^®^ 2810 TR. For *K*_M_ determination, oocytes were incubated in 0.01, 0.03, 0.1, 0.3, 1, 3, or 10 mM l-[ureido-^14^C]citrulline. For determination of leucine uptake in oocytes, the uptake solution was supplemented with 10 µM [4,5-^3^H] leucine (specific activity: 40–60 Ci/mmol, Perkin Elmer, 10 μCi/mL).

### Quantification of Intracellular Amino Acid Content Using High Performance Liquid Chromatography (HPLC)

T cells were activated for 96 h either in the presence of 20 µM arginine or in the complete absence of arginine. Cells were then washed twice in Locke’s solution, supplemented with 2% BSA and incubated in the presence of 1 mM citrulline for 1 or 4 h at 37°C or citrulline was removed immediately to control for unspecific extracellular citrulline attachment. Subsequently, cells were washed 3× with ice-cold Locke’s solution plus BSA before lysis in 200 µL 70% ethanol. Intracellular arginine, ASA, and citrulline levels were determined by HPLC using precolumn derivation and fluorescence detection exactly as published before (29).

### RNA Extraction and Quantitative Real-time Reverse Transcription Polymerase Chain Reaction (qRT-PCR) Analyses

Total RNA was isolated from 5 × 10^6^ human T cells with QIAzol^®^ Lysis reagent (Qiagen, Hilden, Germany) as described by the manufacturer. Transporter mRNA expression was then analyzed by two-step RT-PCR using Taqman hybridization probes as described before (30). Briefly, cRNA was synthesized from 500 ng total RNA with the High Capacity cDNA Reverse Transcription Kit (Applied Biosystems, Darmstadt, Germany) according to the manufacturer’s instructions, followed by qRT-PCR using the StepOnePlus kit (Life Technologies, Carlsbad, CA, USA) and the following primers and probes: hLAT1: ss: 5′-GAAGGGTGATGTGTCCAATCTA, as: 5′-TTCTGTAGGGGTTGATCATTTC, probe: 5′-CAACTTCTCATTTGAAGGCACCAAACTG; h4F2hc: ss: 5′-CTCAGGCAAGGCTCCTGACT as: 5′-GGCAGGGTGAAGAGCATCA, probe: 5′-TGCCGGCTCAACTTCTTCGACTCTAC; β2 microglobulin: ss: 5′-AGCGTACTCCAAAGATTCAGGTT, as: 5′-ATGATGCTCCTTACATGTCTCGAT, probe: 5′-TCCATCCGACATTGAAGTTGACTTACT. All oligonucleotides were purchased by Eurofins MWG Operon, Ebersberg, Germany.

### siRNA-Mediated Knockdown of LAT1 Expression

The siRNA-mediated downregulation of LAT1 expression in human T cells was performed using the Amaxa technology (Lonza, Cologne, Germany). A total of 5 × 10^6^ primary, unstimulated human T cells were washed twice with 1× PBS. T cells were then resuspended in 100 µL human T cell Nucleofector solution (Lonza, Cologne, Germany) before electroporation with 2 µg hLAT1 siRNA (sense: CUCUUUGCCUAUGGAGGAU[dT][dT], Sigma Aldrich) using the Nucleofector™ 2b Device, program V 24 (Lonza). As controls, T cells were either not electroporated or electroporated in the presence of 2 µg non-target RNA (negative control CSR CL 000-005, Eurogentec, S.A.), or without any siRNA. After electroporation, cells were incubated for 5 h in 3 mL prewarmed AIM V^®^ culture medium (Life Technologies, Carlsbad, CA, USA) to recover before further treatment.

### Statistical Analysis

Statistical analyses were performed with the GraphPad Prism software 6. Results are expressed as mean ± SD, if not otherwise indicated, that exhibits mean ± SEM. Statistical differences were calculated using one-way or two-way analysis of variance, followed by the Tukey *post hoc* test, or using *t*-test, as indicated in the figure legends. A PDF file listing *p*-values, obtained in our statistical analyses of comparisons between all distinct experimental groups, is included as Table S1 in Supplementary Material.

## Results

### Citrulline Preserves Human T Cell Proliferation under Limiting Arginine Concentrations

**Figure 1:**
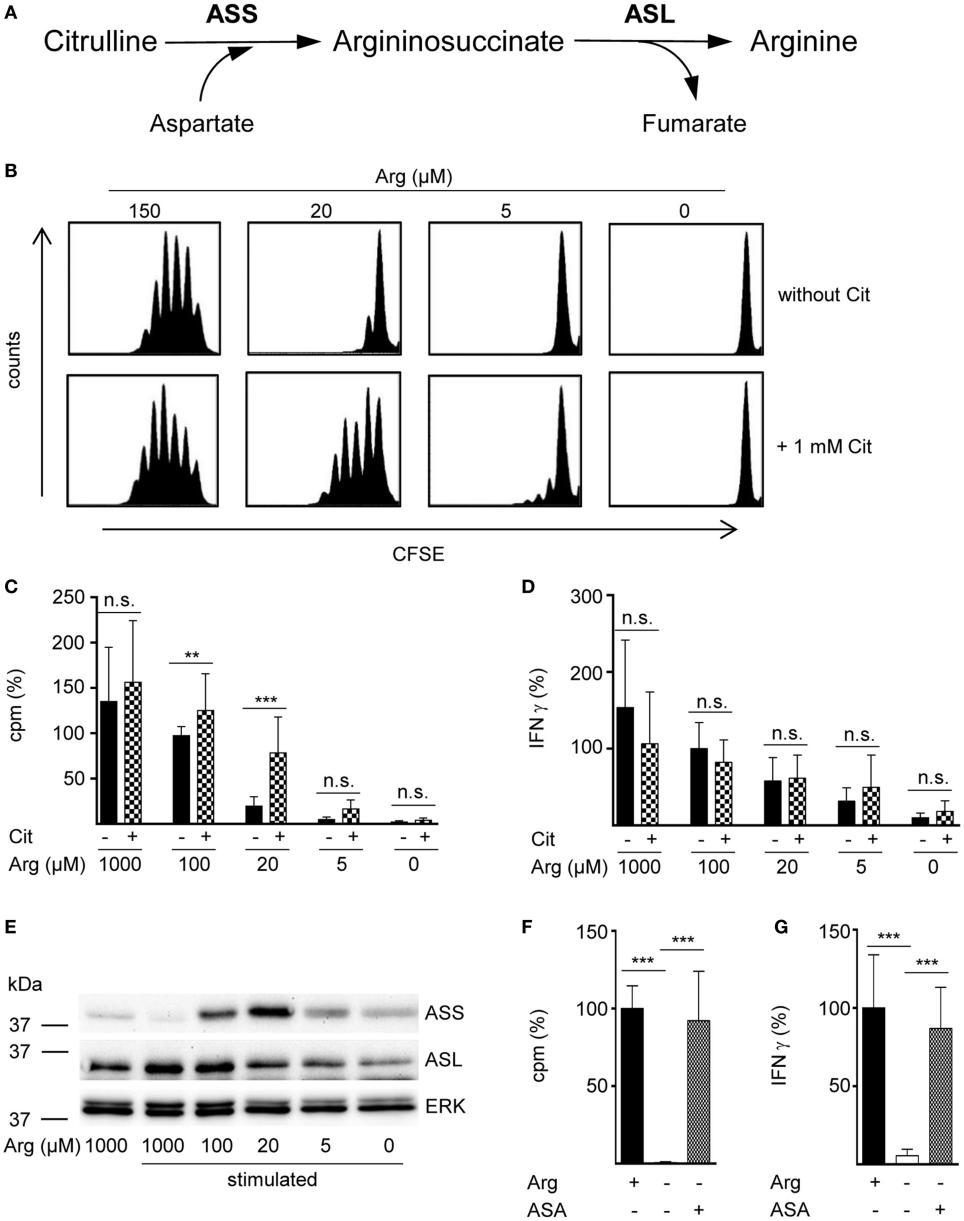
The arginine precursor citrulline preserves human T cell proliferation under low arginine concentrations and this correlates with the induction of argininosuccinate synthase (ASS). **(A)** Scheme showing the two-step conversion of citrulline to arginine by the enzymes ASS and argininosuccinate lyase (ASL). **(B–G)** Primary human CD3^+^ T cells were isolated from blood of healthy donors by negative selection and stimulated with anti-CD3/anti-CD28-tagged beads for 96 h in the presence of the indicated arginine concentrations and either no further supplement or **(B–D)** 1 mM citrulline (Cit) or **(F,G)** 1 mM argininosuccinate (ASA). T cell proliferation was determined **(B)** by 5-(and 6)-carboxyfluorescein diacetate succinimidyl ester staining (one representative experiment of four is shown) and **(C,F)** by the incorporation of [^3^H]thymidine over 16 h (C: *n* = 15–30 from 5 to 10 different donors; F: *n* = 21 from 7 donors). **(D,G)** IFN-γ secretion was detected in T cell supernatants by ELISA (D: *n* = 8–24 from 3 to 8 donors; G: *n* = 9–12 from 4 donors). **(E)** ASS, ASL, and ERK expressions were analyzed in Western blot experiments. Shown is one representative blot of three. Columns represent mean ± SD in percentage of the mean respective value obtained from cells from the same donor stimulated in the presence of 100 µM arginine (C: 12,811 ± 4,173 cpm, D: 1,913 ± 1,421 pg/mL IFN-γ) or 1,000 µM arginine (denoted as Arg+; F: 43,355 ± 29,672 cpm, G: 1,858 ± 2,321 pg/mL IFN-γ). Statistical calculations were performed with one-way analysis of variance and Tukey’s *post hoc* test (***p < 0.001, **p < 0.01, n.s.: *p* ≥ 0.05).

In a variety of cells arginine can be synthesized intracellularly from the neutral amino acid citrulline by the sequential action of the two enzymes ASS and ASL (Figure 1A). However, in the complete absence of arginine, human T cell proliferation cannot be rescued by supplementation of citrulline (16, 23). Since arginine availability *in vivo* can vary substantially under different (patho)physiological circumstances, we tested citrulline substitution at different arginine concentrations. Primary human CD3^+^ T cells were isolated from blood of healthy donors by negative selection, labeled with CFSE, and stimulated with anti-CD3/anti-CD28-tagged beads for 96 h in the presence of 0–150 μM of extracellular arginine with or without supplementation of 1 mM citrulline. T cell proliferation was then analyzed by flow cytometry with loss of CFSE fluorescence corresponding to sequential rounds of cell divisions (Figure 1B). As previously published by us (9, 16) and others (10), human T cell proliferation after CD3/CD28-mediated activation was completely dependent on availability of extracellular arginine: complete and nearly complete cessation of cell proliferation was observed at 0 or 5 µM and at 20 µM arginine, respectively. The effect of citrulline supplementation on T cell proliferation depended on the degree of arginine availability: at 20 µM extracellular arginine, corresponding to a moderate degree of arginine restriction, a pronounced increase in T cell division was always noted. In contrast, at both, very low (here: 5 µM) and physiological (150 µM) arginine concentrations, citrulline did only slightly increase T cell proliferation. When arginine was completely absent, T cells fully remained at rest even when citrulline was substituted, as previously observed by us (16). These findings could also be recapitulated when we monitored T cell proliferation by an alternative assay quantifying [^3^H]thymidine incorporation as a measure of DNA synthesis in the S phase of the cell cycle (Figure 1C). We also included largely supraphysiological extracellular arginine concentrations (1,000 µM) to mimic unrestricted amino acid availability. Again, citrulline did rescue T cell proliferation only when a certain amount of extracellular arginine (optimal: 20 µM) was also present during T cell activation. As the production of the key inflammatory cytokine IFN-γ is critically dependent on arginine availability (11), we analyzed a potential effect of citrulline substitution on its secretion under various arginine conditions (Figure 1D). IFN-γ secretion was correlated with extracellular availability of arginine and was completely shut down in arginine-free conditions. In contrast to the rescue effect of citrulline on T cell proliferation under limiting arginine conditions (Figures 1B,C), we detected no stimulatory effect of extracellular citrulline on the decreased IFN-γ secretion (Figure 1D).

### Preservation of Human T Cell Proliferation by Citrulline under Limiting Arginine Concentrations Correlates with the Induction of ASS

**Figure 2:**
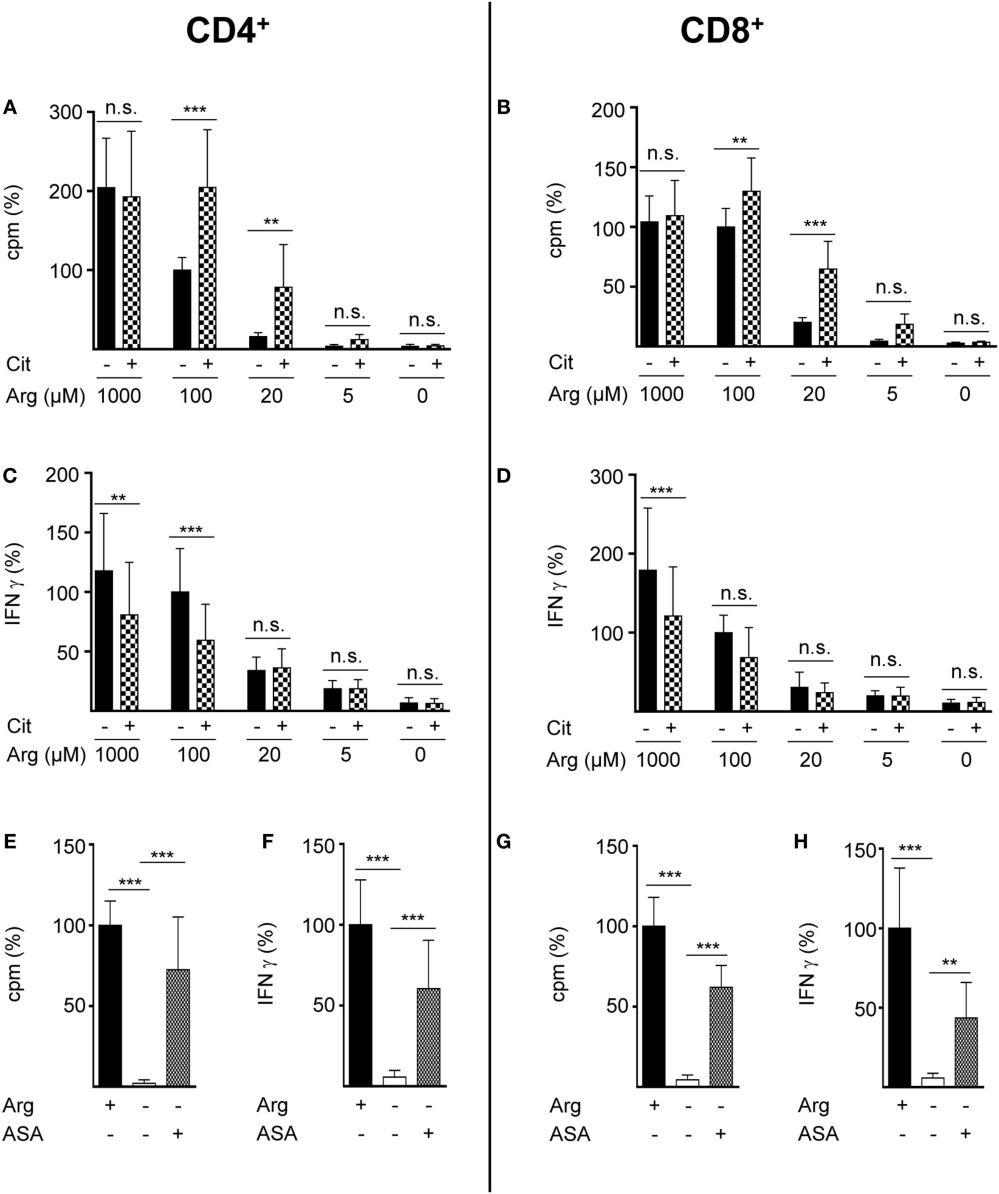
Citrulline supplementation preserves the proliferation of CD4^+^ and CD8^+^ T lymphocytes under low arginine concentrations. CD4^+^ **(A,C,E,F)** and CD8^+^ **(B,D,G,H)** T cells were isolated from blood of healthy donors by positive selection *via* paramagnetic bead technology and stimulated and cultured as in Figure 1. **(A,B,E,G)** T cell proliferation was determined by the incorporation of [^3^H]thymidine over 16 h (*n* = 11–18 from 3 to 6 different donors); **(C,D,F,H)** IFN-γ secretion was detected in T cell supernatants by ELISA (*n* = 10–21 from 4 to 7 different donors). Data are shown as mean ± SD in percentage of the mean respective value obtained from cells of the same batch and stimulated in the presence of 100 µM arginine [**(A)** 11,524 ± 8,138 cpm, **(B)** 11,380 ± 5,223 cpm, **(C)** 1,234 ± 935 pg/mL IFN-γ, **(D)** 490 ± 302 pg/mL IFN-γ] or 1,000 µM arginine [denoted as Arg+; **(E)** 20,736 ± 8,857 cpm, **(F)** 1,238 ± 676 pg/mL IFN-γ, **(G)** 7,771 ± 4,304 cpm, **(H)** 629 ± 467 pg/mL IFN-γ]. Statistical calculations were performed with one-way analysis of variance and Tukey’s *post hoc* test (***p < 0.001, **p < 0.01, n.s.: *p* ≥ 0.05). Cit: citrulline; ASA: argininosuccinate.

Rescue synthesis of intracellular arginine from the precursor citrulline depends on the expression of the two enzymes ASS and ASL (18, 24). A differential expression of either enzyme under varying arginine conditions might account for the arginine concentration-dependent effect of citrulline substitution on T cell proliferation. We, therefore, analyzed the presence of ASS and ASL by Western blot in human T cells activated under various arginine concentrations (Figure 1E). While ASS was not expressed in relevant amounts in activated T cells, when extracellular arginine was not limiting (i.e., at a supraphysiological concentration of 1,000 µM), it was induced upon reduction of arginine, reaching a maximal expression at 20 µM arginine. Upon further reduction of extracellular arginine, ASS expression was again decreased (Figure 1E). In contrast, the expression of the enzyme ASL was constitutive even at supraphysiologically high arginine concentrations and at the complete absence of arginine. Based on these findings we hypothesized that ASA, the intermediate product of the ASS-catalyzed reaction and direct substrate for ASL-mediated arginine synthesis might be able to rescue T cell proliferation at all tested arginine concentrations. When we substituted extracellular ASA to human T cells upon activation we saw full reconstitution of T cell proliferation even in the complete absence of arginine (Figure 1F). Interestingly, ASA substitution also induced complete rescue of IFN-γ secretion in the absence of arginine (Figure 1G), which contrasts the absent effect of citrulline on IFN-γ secretion (Figure 1D). Next, we studied the influence of citrulline on proliferation and IFN-γ secretion under the different conditions of arginine availability in highly purified CD4^+^ and CD8^+^ human T cell subsets. The two T cell subpopulations did not differ essentially in their response to citrulline substitution: proliferation of both CD4^+^ (Figure 2A) and CD8^+^ (Figure 2B) T cells was rescued by citrulline supplementation to various degrees only when arginine was moderately reduced (20–100 µM), while at lower arginine concentrations, especially in the complete absence of arginine, no significant increase in T cell proliferation could be detected. At supraphysiological arginine concentrations (1,000 µM), citrulline did not further increase proliferation (Figures 2A,B). No rescue of activation-induced CD4^+^ (Figure 2C) and CD8^+^ (Figure 2D) T cell IFN-γ secretion was detectable upon citrulline substitution at all tested arginine concentrations, which corresponds to the results of the experiments with bulk CD3^+^ T cells (Figure 1D). In contrast, in arginine-sufficient conditions, citrulline even had an inhibitory effect on IFN-γ secretion (Figures 2C,D). We then also tested the substitution of ASA to bypass the necessity for ASS expression in both CD4^+^ and CD8^+^ human primary T cells. Upon substitution of ASA we again saw significant reconstitution of CD4^+^ (Figure 2E) and CD8^+^ (Figure 2G) T cell proliferation as well as IFN-γ secretion (Figures 2F,H, respectively) even in the complete absence of extracellular arginine.

### Activated Human T Cells Take Up Citrulline, but Conversion to Intracellular Arginine Depends on Availability of Low Amounts of Extracellular Arginine

**Figure 3:**
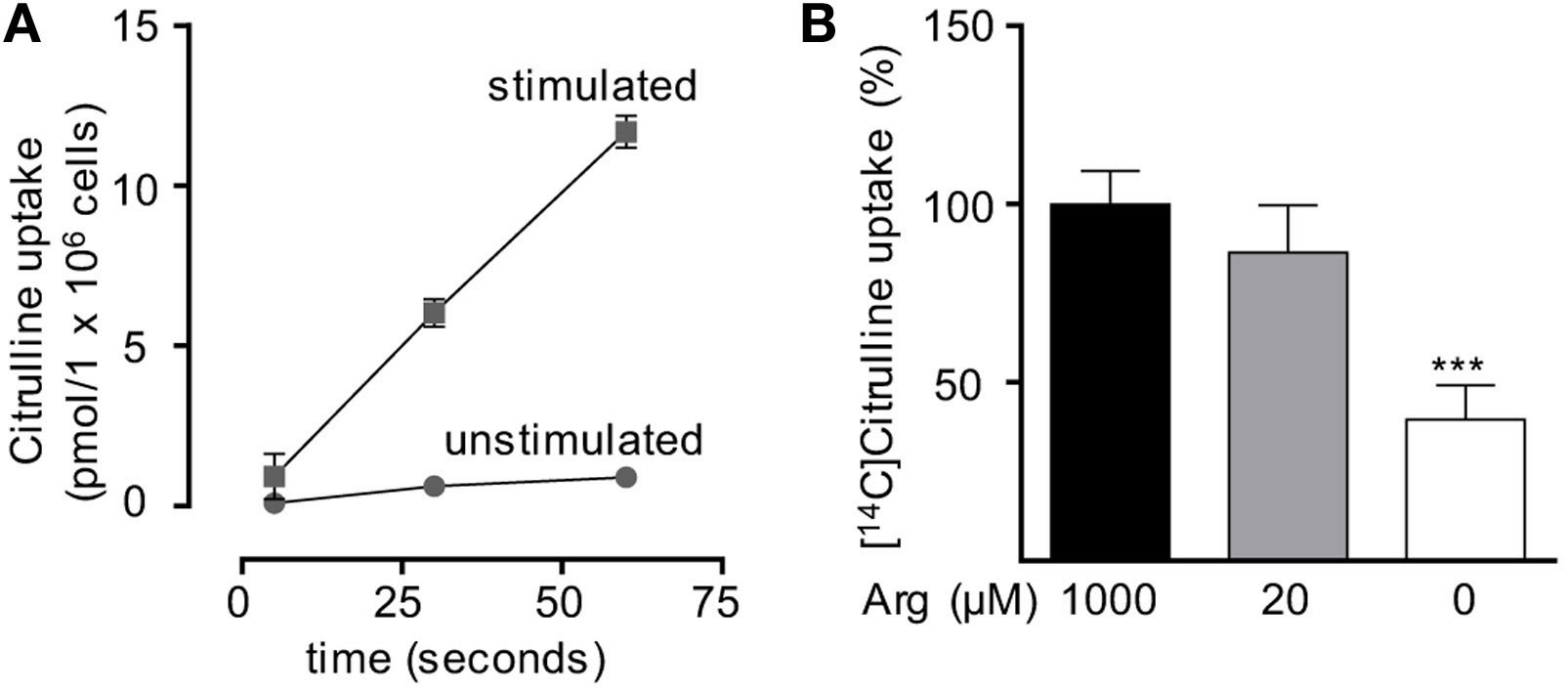
Citrulline uptake is induced upon T cell stimulation. Primary human CD3^+^ T lymphocytes were isolated from blood of healthy donors by negative selection. **(A)** Time-dependent uptake of 20 µM [^14^C]citrulline into T cells stimulated in the presence of 20 µM arginine for 24 h with anti-CD3/anti-CD28-tagged particles (squares) or left unstimulated (circles). Each data point represents the mean ± SD of three cell aliquots from one blood donor. Comparable results were obtained with cells from two other blood donors. **(B)** T cells were stimulated with anti-CD3/anti-CD28-tagged particles for 24 h in the presence of the indicated arginine concentrations. Citrulline uptake was then measured after incubation with 20 µM [^14^C]citrulline for 30 s. Data represent mean ± SD in percentage of the values obtained with cells from the same donor and stimulated in the presence of 1,000 µM arginine (10.21 ± 8.4 pmol arginine/10^6^ cells, *n* = 8–9 from three different donors). Statistical calculations were performed with one-way analysis of variance and Tukey’s *post hoc* test (***p < 0.001).

**Figure 4:**
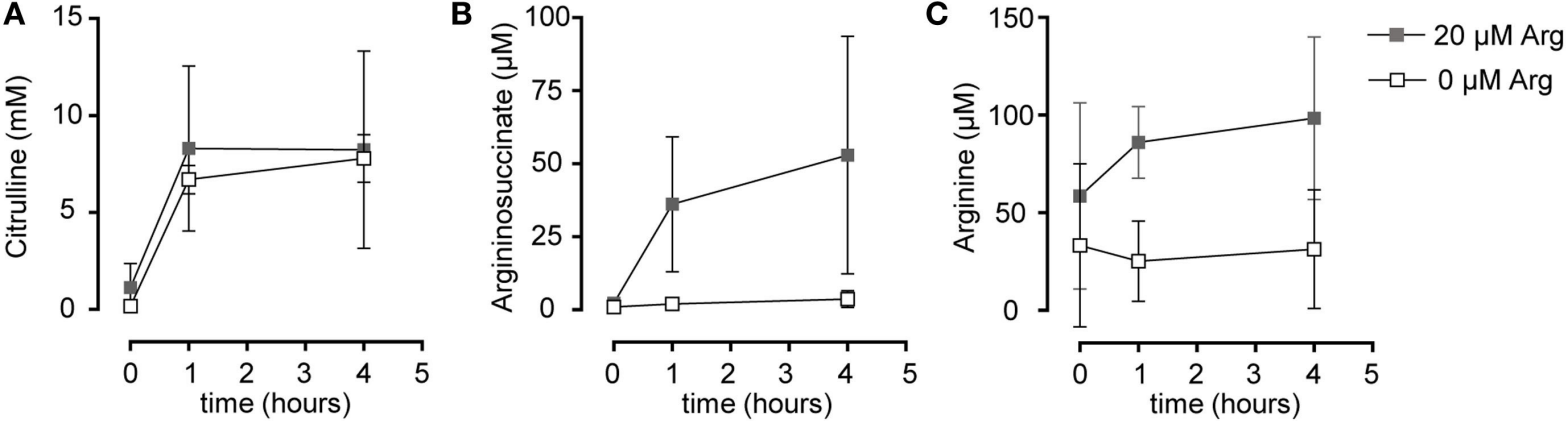
Stimulated T cells convert citrulline to arginine only when cultured in the presence of 20 µM arginine, but not in its complete absence. Primary human CD3^+^ T lymphocytes were isolated from blood of healthy donors by negative selection and stimulated in the presence of 20 µM arginine or in the complete absence of arginine with anti-CD3/anti-CD28-tagged beads for 96 h. Citrulline at a final concentration of 1 mM was then added to the cells and they were either incubated for further 1 or 4 h or citrulline was removed immediately (*t* = 0 h). Intracellular concentrations of **(A)** citrulline, **(B)** argininosuccinate, and **(C)** arginine were quantified by high performance liquid chromatography. Statistical calculations were performed with two-way analysis of variance (*n* = 3).

Based on our functional studies (Figures 1 and 2), we concluded that activated human T cells can take up citrulline and metabolize it further to arginine, but this process depends on the availability of a minimum amount of extracellular arginine. We, therefore, wanted to further validate this hypothesis by directly measuring citrulline uptake and intracellular metabolic products of the ASS- and ASL-mediated enzymatic reactions upon T cell activation. We first analyzed, if citrulline uptake by T cells is induced by activation. Human T cells were stimulated for 24 h with anti-CD3/anti-CD28-tagged beads in the presence of 20 µM arginine, a setting, in which citrulline substitution can rescue deficient T cell proliferation (Figures 1B,C and 2A,B). These cells as well as unstimulated T cells were then shortly exposed to [^14^C]citrulline and uptake was quantified. While unstimulated T cells did not show relevant uptake of citrulline, preactivation *via* the TCR and CD28 costimulation clearly induced vigorous citrulline transport (Figure 3A). Quantitatively, the citrulline uptake capacity upon T cell activation was in the same order of magnitude as the arginine uptake capacity previously observed by us (16). The inability of extracellular citrulline to (i) further increase T cell proliferation under supraphysiological arginine availability or (ii) to rescue T cell proliferation in the complete absence of extracellular arginine (Figures 1B,C and 2A,B) might be due to an inability of the activated T cells to generate citrulline transport capacity, e.g., because of deficient transporter synthesis or impaired translocation to the membrane. We tested this by activating T cells in the presence of 1,000, 20, and 0 µM arginine for 24 h and subsequent quantification of citrulline uptake. These experiments demonstrated that citrulline transport capacity is comparably induced under supraphysiological (1,000 µM) and moderately limiting (20 µM) arginine availability (Figure 3B). Stimulation in the complete absence of arginine induced significantly less citrulline transport capacity (60.2 ± 15.2% relative to 1,000 µM arginine, Figure 3B). To analyze if this reduction may contribute to the complete absence of citrulline function in T cells under the same conditions, we stimulated T cells either in the absence or in the presence of 20 µM arginine for 96 h and then supplemented citrulline for 1 or 4 h. The resulting intracellular citrulline, ASA, and arginine concentrations were quantified by HPLC (Figure 4). Intracellular citrulline concentrations were largely increased upon external supplementation under both conditions (*p* = 0.001). Further citrulline metabolism, however, was completely different between T cells stimulated in the presence of 20 µM arginine or in its absence: in the context of citrulline supplementation and the presence of 20 µM extracellular arginine, the intracellular concentrations of ASA and also arginine rose steeply. In contrast, in the absence of extracellular arginine no further metabolism of intracellular citrulline to ASA (Figure 4B), or arginine (Figure 4C) could be detected (20 µM extracellular arginine versus 0 µM extracellular arginine: *p* = 0.0088 for intracellular ASA and *p* = 0.0096 for intracellular arginine).

### Citrulline Is Transported into Human T Cells *via* L-Type Amino Acid Transporter 1 (LAT1)

**Figure 5:**
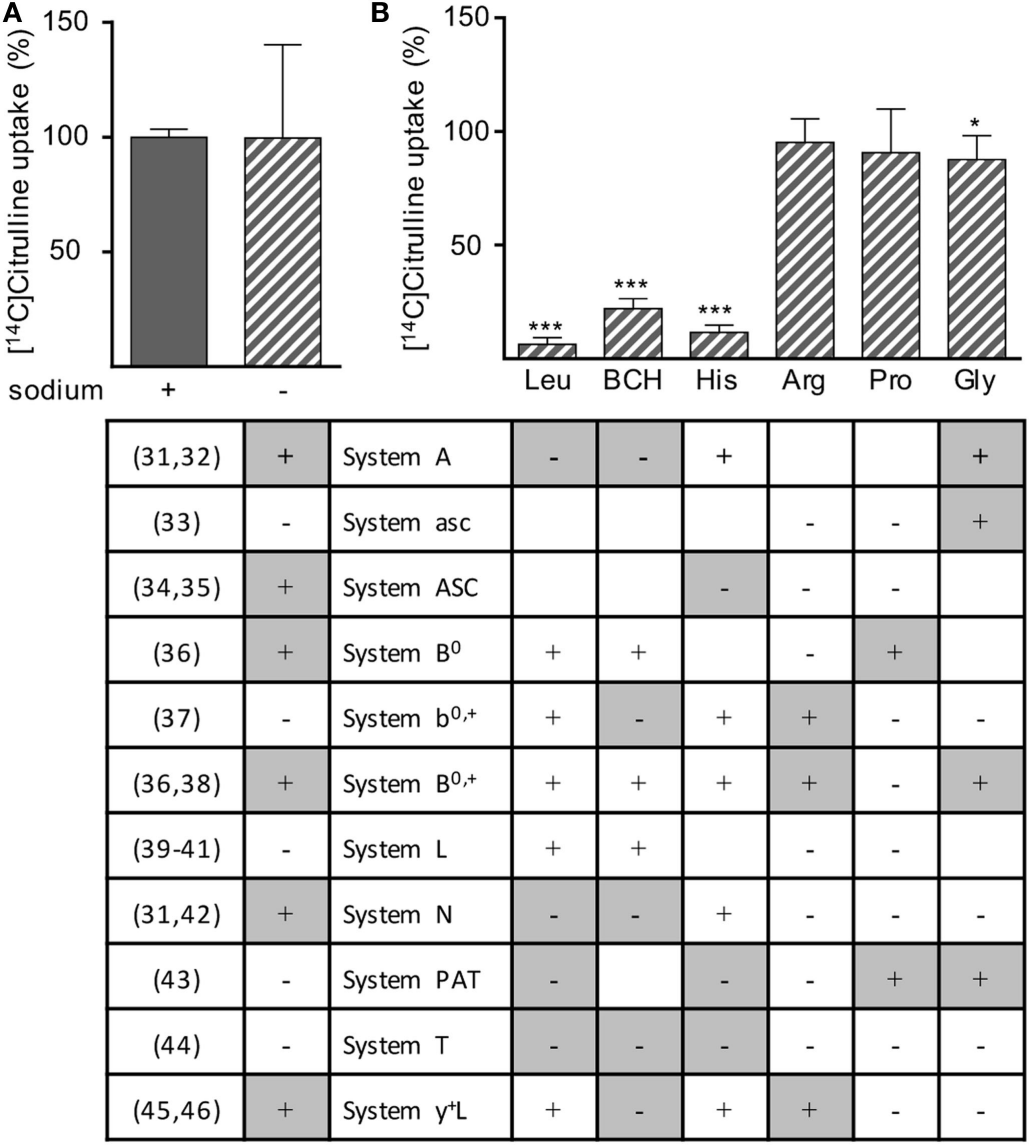
Citrulline uptake into human T lymphocytes seems to be mediated by L-type amino acid transporter 1 (LAT1). Primary human CD3^+^ T lymphocytes were isolated from blood of healthy donors by negative selection and stimulated for 24 h in the presence of 20 µM arginine with anti-CD3/anti-CD28-tagged particles. The uptake of 20 µM [^14^C]citrulline was then determined over 30 s in the presence or absence of **(A)** sodium or **(B)** in the presence of 1 mM leucine (Leu), 2-aminobicyclo-(2,2,1)-heptane-2-carboxylic acid (BCH), histidine (His), arginine (Arg), proline (Pro), or glycine (Gly). Data are expressed as percentage of the mean value obtained with the respective control cells (without any competitive amino acid and in the presence of sodium), **(A)** 5.0 ± 1.0 pmol citrulline/10^6^ cells, *n* = 12 from 4 donors; **(B)** 7.4 ± 5.7 pmol citrulline/10^6^ cells, *n* = 9 from three donors. Statistical calculations were performed with two-tailed *t* test between competitor-treated and respective control cells (***p < 0.001,*p < 0.05). The table below the respective experimental conditions denotes the expected results according to the cited published references (left column), i.e., if transported *via* the respective system. +: inhibition of citrulline uptake expected; −: no inhibition of citrulline uptake expected. Color coding of table: experimental results were classified as compatible (white) or not compatible (gray) with the respective transport system; empty cells: interpretation of the results equivocal.

Various different transporter systems can mediate the transport of neutral amino acids *via* the plasma membrane: system A (31, 32), asc (33), ASC (34, 35), B^0^ (36), b^0,+^ (37), B^0,+^ (36, 38), L (39–41), N (31, 42), PAT (43), T (44), and y^+^L (45, 46). To determine which transport system, respectively, which transporter is responsible for citrulline uptake into activated human T cells, we stimulated human T cells for 24 h with anti-CD3/anti-CD28-tagged beads and measured the uptake of [^14^C]citrulline into these cells in the presence or absence of sodium (Figure 5A) and in the presence of BCH, an amino acid considered a model substrate for system L (39–41) and selected amino acids, known to specifically inhibit certain defined transport systems (Figure 5B). The table in Figure 5 delineates if inhibition of the respective transport system by the omission of Na^+^ or by the respective competing amino acid is expected (+) or not (−), as deduced from the cited literature. Experimental results were classified as compatible (white) or not compatible (gray) with the respective transport system. The experiments demonstrated that citrulline uptake into activated T cells is mediated *via* a sodium-independent transport system (Figure 5A), excluding all Na^+^-dependent transporter systems for neutral amino acids, namely A, ASC, B^0^, B^0,+^, N, and y^+^L. Citrulline transport was inhibited by leucine, BCH, and histidine, but not by arginine, proline, or glycine, further excluding the systems mentioned above as well as the Na^+^-independent system asc, b^0,+^, PAT, and T (Figure 5B). This pattern of competitors/non-competitors with citrulline transport in T cells is uniquely compatible with amino acid transport *via* system L (Figure 5). Within this system, four different transporters are known: LAT1-4. They differ in their substrate recognition: while transport by all LAT proteins can competitively be inhibited by leucine and BCH, only LAT1 and LAT2 are also inhibited by histidine (39–41), as detectable in our transport assays (Figure 5). LAT2 also demonstrates transport activity for glycine (39) that inhibited citrulline transport in T cells significantly, but only by 12.4 ± 10.6% (Figure 5). Our transport assays, therefore, demonstrate that activated human primary T cells take up citrulline mainly *via* a transporter with the transport characteristics of LAT1 (SLC7A5), a member of system L within the heteromeric amino acid transporter family of proteins (13, 14).

**Figure 6:**
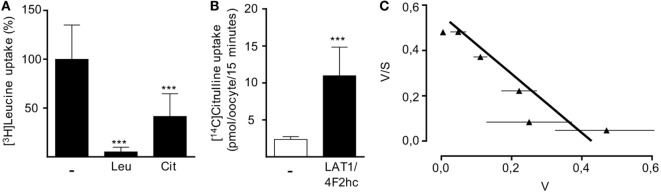
LAT1 mediates citrulline uptake. *Xenopus laevis* oocytes were injected with cRNA encoding for LAT1 and the associated glycoprotein 4F2hc and incubated for 2 days to allow for protein expression. Non-injected oocytes served as controls. **(A)** The uptake of 10 µM [^3^H]leucine over 15 min was then assessed in the presence or absence of 2 mM unlabeled leucine (Leu) or citrulline (Cit). Basal [^3^H]leucine uptake measured in control oocytes was subtracted from values obtained with LAT1/4F2hc cRNA-injected oocytes. Data are expressed as percentage of the mean value obtained from leucine uptake into oocytes without any competitive amino acid (11.3 ± 7.2 pmol leucine/oocyte/15 min; *n* = 16–28). **(B)** The uptake of 20 µM [^14^C]citrulline over 15 min in control oocytes and LAT1/4F2hc cRNA-injected oocytes was determined (mean ± SD, *n* = 11–12). Statistical calculations were performed with one-way analysis of variance and Tukey’s *post hoc* test **(A)** and with *t*-test **(B)** (***p < 0.001). **(C)** Eadie–Hofstee graphical representation of concentration-dependent uptake of [^14^C]citrulline over 15 min. Basal [^14^C]citrulline uptake in control oocytes was subtracted from values obtained with LAT1/4F2hc cRNA-injected oocytes (mean ± SEM, *n* = 5–7 oocytes from one frog). Similar results were obtained with oocytes from two other animals.

In order to directly examine the ability of LAT1 to transport citrulline, we injected *X. laevis* oocytes with cRNA encoding for LAT1 and the associated glycoprotein 4F2hc. After 48 h, LAT1 protein was detectable by Western blot in the injected oocytes (data not shown). Since the large neutral amino acid leucine is a well-known transport substrate for LAT1, we first analyzed if coapplication of citrulline would be able to competitively inhibit leucine uptake in LAT1-injected frog oocytes (Figure 6A). The uptake of 10 µM [^3^H]leucine was indeed inhibited by 58.6 ± 23.2% in the presence of 2 mM citrulline, while a 94.3 ± 4.7% inhibition was observed in the presence of the same concentration of non-radioactive leucine (Figure 6A), suggesting a low affinity of LAT1 for citrulline. To directly prove that citrulline is a transport substrate for LAT1, uptake of [^14^C]citrulline was then quantified in non-injected control oocytes and in the LAT1/4F2hc-expressing oocytes: expression of the heteromer was associated with a 4.6 ± 1.6-fold increased citrulline import (Figure 6B). The apparent substrate affinity of LAT1 for citrulline was then determined at [^14^C]citrulline concentrations between 0.01 and 10 mM. The citrulline concentrations at which half-maximal transport rates were reached (apparent *K*_M_) were calculated by fitting the data according to the Eadie–Hofstee equation after subtraction of the values obtained with non-injected oocytes (Figure 6C). The apparent *K*_M_ value obtained in these experiments was 0.79 ± 0.08 mM, confirming citrulline as a substrate of LAT1 that is recognized with low affinity.

### LAT1 and 4F2hc Are Induced when Primary Human T Cells Are Activated Even under Arginine Starvation and Are Necessary for Citrulline Uptake

**Figure 7:**
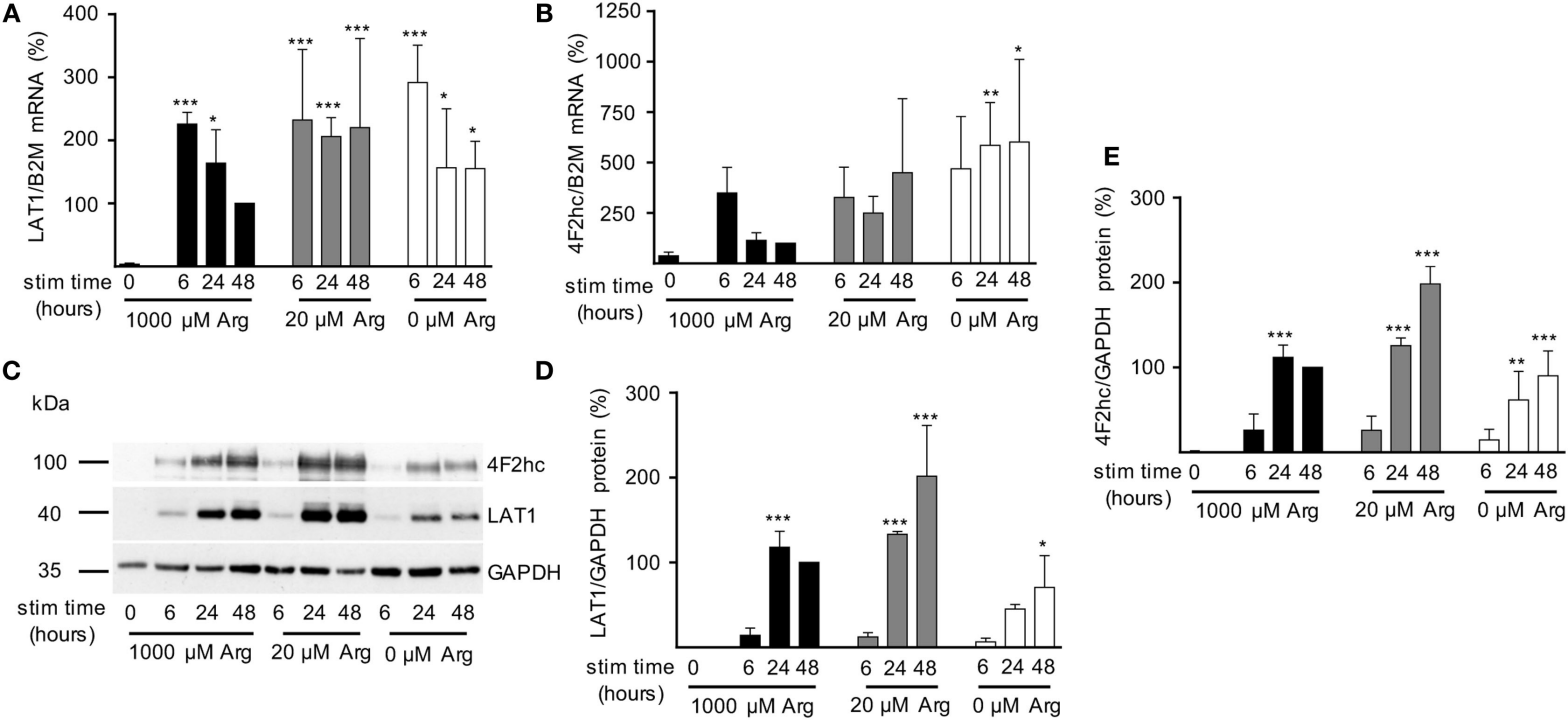
Expression of LAT1 and 4F2hc is induced upon T cell stimulation even under limiting arginine concentrations. Primary human CD3^+^ T lymphocytes were isolated from blood of healthy donors by negative selection. T cells were stimulated in medium containing 1,000, 20, or 0 µM arginine for 6, 24, or 48 h or left unstimulated (0 h). LAT1 **(A)** and 4F2hc **(B)** mRNA expression levels were quantified by quantitative real-time reverse transcription polymerase chain reaction. B2M (β2-microglobulin) was used for relative measurements. Data are expressed as percentage of the respective LAT1/B2M or 4F2hc/B2M ratio obtained in the same experiment for cells stimulated for 48 h in 1,000 µM arginine [mean ± SD, **(A)** *n* = 4–5, **(B)** *n* = 3–4]. **(C,D,E)** LAT1, 4F2hc, and glyceraldehyde 3-phosphate dehydrogenase (GAPDH) protein expressions were determined by Western blot. **(C)** Representative blot, **(D)** quantification of LAT1, and **(E)** 4F2hc protein. Data are expressed as percentage of the respective LAT1/GAPDH or 4F2hc/GAPDH ratio obtained in the same experiment for cells stimulated for 48 h in 1,000 µM arginine (mean ± SD, *n* = 3). For statistical analysis each time point of stimulated T cells was compared to unstimulated control cells (0) (***p < 0.001, **p < 0.01,*p < 0.05), two-way analysis of variance with Tukey’s posttest.

**Figure 8:**
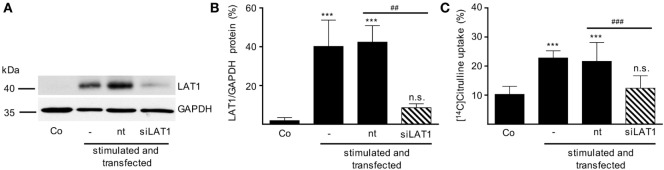
Inhibition of LAT1 expression in stimulated T lymphocytes suppresses activation-induced citrulline transport. Primary human CD3^+^ T lymphocytes were isolated from blood of healthy donors by negative selection. CD3^+^ T cells were electroporated in the absence of RNA (−) or transfected with non-target RNA (nt) or LAT1 siRNA (siLAT1). After 4 h, cells were stimulated with anti-CD3/anti-CD28-coupled beads for 24 h. Non-stimulated T cells were included as further controls (Co). **(A,B)** LAT1 and glyceraldehyde 3-phosphate dehydrogenase (GAPDH) protein expression was determined by Western blot; **(A)** representative blot, **(B)** quantification of three independent experiments. Data are expressed as percentage of the respective LAT1/GAPDH ratio obtained in the same experiment for cells stimulated for 24 h without any transfection (mean ± SD, *n* = 3). **(C)** The uptake of 20 µM [^14^C]citrulline over 30 s was evaluated and expressed as percentage of values obtained with 24 h-stimulated, non-transfected cells (19.56 ± 9.49 pmol citrulline/10^6^ cells, mean ± SD, *n* = 8–12 from 3 to 5 donors). Statistical analyses were performed using one-way analysis of variance with Tukey’s *post hoc* test (*** or ^###^*p* < 0.001, ** or ^##^*p* < 0.01, n.s.: *p* ≥ 0.05).

After having demonstrated that (i) activated human T cells upregulate a transport system for citrulline (Figures 3 and 4), (ii) the transport characteristics of this system are compatible with the LAT1 transporter (Figure 5), and (iii) the LAT1 protein is indeed able to transport citrulline (Figure 6), we next analyzed mRNA and protein expression of LAT1 and the associated glycoprotein 4F2hc in T cells upon activation at various time points and under different arginine conditions. Corresponding to the very low citrulline transport activity in resting human T cells we saw no relevant expression of LAT1 (Figure 7A) and 4F2hc (Figure 7B) mRNA in unstimulated T cells. Upon anti-CD3/anti-CD28-mediated activation, there was a pronounced induction of LAT1 and 4F2hc mRNA at 6 h in the context of all tested arginine concentrations for both mRNAs. While LAT1 and 4F2hc mRNA levels dropped later on under optimal arginine availability (1,000 µM), they were persistently upregulated in the presence of 20 µM arginine. LAT1 and 4F2hc proteins were also induced in the activated T cells (Figures 7C–E). Here, transporter protein started to appear at 6 h, with a strong increase at 24 h. At 48 h, both proteins reached a plateau under 1,000 µM arginine and even continued to increase under arginine starvation. While LAT1 (Figure 7A) and 4F2hc mRNA (Figure 7B) were also highly induced in the complete absence of arginine, protein expression levels for both transporter components were reduced compared to the conditions with 1,000 and 20 µM extracellular arginine (at 24 h LAT1 and 4F2hc were reduced to 39.0 ± 9.2% and 57.4 ± 37.2% compared to 1,000 µM arginine, *p* = 0.0147 and *p* = 0.0499, respectively). This may be due to the severe shutdown of *de novo* protein synthesis that is a consequence of amino acid depletion, but it nicely corresponds to the reduced citrulline uptake (Figure 3B) upon T cell stimulation in the absence of extracellular arginine. Finally, in order to causally prove the necessity of LAT1 expression for the citrulline import in activated human T cells, we transfected primary human T cells with LAT1 siRNA. This led to a significant (79.0 ± 7.3% versus control transfection with non-target RNA) inhibition of anti-CD3/anti-CD28-mediated induction of LAT1 transporter protein (Figures 8A,B). These LAT1-suppressed T cells demonstrated a significant 44.4 ± 9.3% reduction in activation-induced citrulline uptake. Their citrulline transport rates were not significantly different from unactivated primary human T cells (Figure 8C).

## Discussion

Our study has been set out to define the parameters of a potential functional rescue of primary human T cell proliferation by citrulline supplementation in the setting of restricted arginine availability that occurs during inflammation when myeloid cells express and secrete the arginine degrading enzyme arginase. In contrast to earlier studies, including our own (16, 23), that failed to see such a rescuing effect of citrulline in the complete absence of extracellular arginine, we found here that citrulline can fully support restricted proliferation of T cells under limiting arginine concentrations (20 µM). The inability of citrulline to increase T cell proliferation in the complete absence of extracellular arginine correlated not only with the absence of relevant amounts of ASS (Figure 1E) and consequently with the absence of downstream citrulline metabolism toward arginine (Figures 4B,C), even though citrulline was very efficiently translocated into the cell (Figure 3B) reaching high intracellular levels (Figure 4A). In older literature it has already been demonstrated that resting human T cells do not express ASS (25), while elevated ASS activity has been found in long-term cultures of lymphoblasts in the absence of arginine and in the presence of citrulline (25, 47, 48) and in activated T cells of patients with systemic lupus erythematosus (26). ASS induction and ASS-dependent citrulline-mediated rescue of cellular proliferation is well known for various tumor cell entities (49). It has also been described in human adult leukemic T cell lines (22, 25). However, these cells are permanently in cell cycle and are, therefore, not an adequate model to study T cell physiology and complex activation processes. We, therefore, analyzed if activated primary human T cells respond to arginine depletion by induction of ASS and ASL and if exogenous citrulline supplementation can rescue T cell proliferation under arginine limiting conditions. We found that *de novo* synthesis of ASS in activated human primary T cells is based on limited amounts of extracellular arginine. ASS was induced neither at supraphysiological arginine concentrations nor in the complete absence of arginine (Figure 1E). Also, the presence of citrulline did not significantly alter the expression level of ASS (data not shown). Correlating with ASS induction, T cell proliferation was rescued at limiting arginine concentrations by the addition of citrulline (Figures 1B,C), which was metabolized intracellularly *via* ASS and ASL to arginine (Figure 4). No rescue of T cell proliferation by citrulline was seen in the complete absence of arginine (Figures 1B,C). Our findings are compatible with a scenario in which ASS expression in stimulated human T cells is based both on an activated sensor of arginine deficiency and on the concurrent availability of still enough residual arginine for initial *de novo* ASS protein translation. This is in contrast to human Jurkat T cells (22) and murine primary T cells (23), which both show expression of ASS in the complete absence of exogenous arginine. It remains to be analyzed, how expression of ASS is regulated in activated human T cells in the context of varying arginine concentrations. In general, ASS expression is regulated both transcriptionally and posttranscriptionally (18, 50). In various cancer entities, ASS promoter methylation prevents ASS expression. HIF-1α and c-myc are important transcription factors for the extrahepatic regulation of ASS (50), and the interplay between HIF-1α, c-myc, and Sp4 determines the expression of ASS in melanoma cells in the context of varying degrees of arginine availability (51). Murine macrophages upregulate ASS expression early on in an infection so that later on, under arginine starvation upon extended periods of inflammation, the arginine-recycling enzyme is already expressed (21). It remains to be determined, if a similar priming for ASS expression can be achieved in human T cells. The reason for the differential effect of citrulline on T cell proliferation versus IFN-γ secretion (Figure 1) remains unclear. Supplementation of ASA, bypassing the need for ASS, leads to reconstitution of both proliferation and IFN-γ secretion. One potential explanation for these discrepancies might be that different levels of intracellular arginine concentrations are necessary for cell cycle progression and cytokine synthesis. However, this seems unlikely because our earlier work indicates that IFN-γ production is less sensitive to arginine limitation than cell proliferation (9, 11, 16). Because LAT1 functions as an obligatory exchanger, one can assume that citrulline supplementation will deplete other intracellular LAT1 substrates, e.g., many essential neutral amino acids. In both murine and human T cells, inhibition of LAT1 preferentially inhibits cytokine synthesis rather than proliferation (52, 53). We, therefore, hypothesize that the lack of citrulline to reconstitute IFN-γ production may rather be due to this citrulline-mediated depletion of intracellular neutral amino acids.

It is increasingly becoming clear that amino acid transporters are key gatekeepers in activated T cells. Expression of CAT1 [SLC7A1 (16)], ASCT2 [SLC1A5 (54)], LAT1 [SLC7A5 (52, 53)], and SNAT1 and 2 [SLC38A1 and A2 (55)] are induced by activation of T cells *via* the TCR and costimulation. Upon expression, these amino acid transporters increase the T cell import of amino acids like arginine *via* hCAT1 (16), alanine *via* ASCT2 (54), leucine *via* LAT1 (52, 53), and glutamine *via* SNAT1 and 2 (55). The intracellular availability of these amino acids profoundly dictates T cell metabolism and functions like proliferation and/or cytokine synthesis (16, 52–55). It is difficult to define the specific role of individual amino acid transporters for T cell function in a (patho-) physiological context *in vivo*, since inhibition of one transporter subtype entails complex alterations of cellular metabolism (52, 54). Nevertheless, specific amino acid transporters have already been shown to be involved in the regulation of T cell function in infection (55, 56) and autoimmunity (54). We demonstrate here that LAT1 has an additional function in supplying citrulline under arginine depletion.

We showed that citrulline uptake in activated arginine-deprived human T cells occurs largely *via* the heteromeric system L amino acid transporter LAT1/4F2hc. Different cell or tissue types use different neutral amino acid transport systems for uptake of citrulline (27, 57). As found here for T cells, intestinal enterocytes take up the amino acid *via* system L. However, they also take up citrulline *via* the broad scope transporter systems B^0,+^ and b^0,+^ (58) that are hardly detectable in activated human primary T cells (16). In piglet pulmonary endothelial cells, citrulline transport occurs *via* the Na^+^-dependent SNAT1 (59). In contrast, our data do not suggest that citrulline is a substrate for human SNAT1. This transporter is expressed in activated human T cells (55). However, we found only minimal suppression of citrulline transport by glycine and no suppression by the omission of sodium. Our previous results suggest that citrulline is also a substrate for the system N transporter SN1 [SNAT3 or SLC38A3 (60)], but no SN1 expression has been reported in activated human T cells. Even in the complete absence of arginine, there was still significant LAT1 and 4F2hc protein expression (Figure 7) and citrulline import (Figure 3B). LAT1 transports amino acids as obligatory exchanger, coupling the influx of an extracellular substrate to the efflux of an intracellular amino acid (13, 14, 29, 61). External supplementation of 1 mM citrulline to activated T cells led to much higher intracellular concentrations (Figure 4A). This is compatible with a concentrative transport activity. Therefore, the ability of citrulline to enhance or reconstitute T cell proliferation is clearly not limited by citrulline uptake, but depends on the degree of ASS expression (Figure 1E) and ASS-mediated metabolism to ASA and arginine (Figures 4B,C).

Several interesting questions remain regarding the effects of citrulline for activated T cells. Recently, a detailed metabolomic study demonstrated that arginine supplementation with resultant increased arginase II-mediated metabolism leads to increased oxidative phosphorylation with enhanced generation of T cell memory (6). It remains to be determined if, under limiting arginine, the importance of citrulline substitution is solely based on its regeneration of arginine *via* ASS and ASL or if it is partly due to a feeding into the anaplerotic Krebs cycle *via* the ASL reaction product fumarate, fulfilling broader functions as intermediary metabolic product (5). While we have shown the rescue effect on T cell proliferation, it remains to be determined if citrulline substitution can drive or influence T cell polarization. Glutamine uptake *via* the transporter ASCT2 is non-redundantly important for Th1/Th17 differentiation, but dispensable for T cell proliferation (54). In cancer cells, leucine uptake *via* LAT1 has been shown to be connected to glutamine uptake *via* ASCT2 (62) and in murine T cells, Glut1 glucose transporter expression is functionally coupled to glutamine uptake *via* ASCT2 (54). Does LAT1 entertain similar interconnections to different transporters in human primary T cells and how are these affected by arginine starvation and citrulline supplementation?

Finally, our current data might help to design novel treatment strategies for unwanted immunosuppression. The micromilieu of an inflammatory tumor stroma is characterized by a pronounced exchange of nutrients, cells, and lymphatic liquid, so that a complete arginine depletion by arginase is likely the exception and suboptimal arginine concentration with associated T cell suppression [EC_50_: 20 µM (63)] is the clinically relevant pathophysiological scenario (5, 64). In this context, reconstitution of T cell proliferation by citrulline supplementation is a very promising approach, which potentially could help to overcome immunosuppression in inflammatory diseases and cancer. Arginase-mediated arginine depletion might be addressed pharmacologically by inhibition of arginase, arginine supplementation, or supply of the precursor citrulline for intracellular arginine synthesis. Arginase inhibitors have been successfully used to reconstitute immune function both *in vitro* (9) and *in vivo* (65), and an arginase inhibitor is already in phase I clinical testing (ClinicalTrials.gov:NCT0290391). Various animal studies as well as clinical trials have analyzed the influence of oral or parenteral arginine supplementation on different parameters of the immune system. In summary, no clear-cut reproducible advantage regarding immune functions or clinical outcome emerged so far and the optimal indication for use of arginine as immunonutrition is still not clear (64, 66). In the context of arginase-mediated arginine depletion, substitution of arginine is likely of limited efficacy due to its enzymatic hydrolysis by liberated (9) or cell-bound (67) arginase. Citrulline is not metabolized by arginase and no significant intestinal degradation of citrulline takes place. In a murine model of arginase-mediated arginine depletion, citrulline supplementation triggered increased NO synthesis and microcirculatory flow in contrast to the treatment with arginine only (68). Human citrulline pharmacokinetics has already been investigated (27, 57, 68). Importantly, the oral administration of citrulline significantly increased arginine plasma concentrations without relevant side effects in a double-blind, placebo-controlled study (28).

In summary, our data reveal novel important aspects of T cell activation under arginine deprivation and demonstrate the requirements for and potential of citrulline to be used as a precursor amino acid for T cell endogenous arginine synthesis with consecutive exit from the immunosuppressed state.

## Ethics Statement

This study was carried out in accordance with the recommendations of the Rhineland Palatine Medical Association Ethics Committee with written informed consent from all subjects. All subjects gave written informed consent in accordance with the Declaration of Helsinki. The protocol was approved by the Rhineland Palatine Medical Association Ethics Committee.

## Author Contributions

AW, MK, NL, CL, AH, JR, CH, RC, EC, and MM contributed to the conception and/or design of the work; AW, MK, NL, CL, AH, JR, CH, and RC contributed to data acquisition; AW, MK, JR, CH, EC, and MM performed data analysis and interpretation; AW, EC, and MM drafted the manuscript; AW, MK, NL, CL, AH, JR, CH, RC, EC, and MM revised the manuscript; AW, EC, and MM finalized the article. All the authors have read and approved the final manuscript and agreed to be accountable for all aspects of the work.

## Conflict of Interest Statement

The authors declare that the research was conducted in the absence of any commercial or financial relationships that could be construed as a potential conflict of interest.
