# Supplementary material for: Reconstitution of T Cell Proliferation under Arginine Limitation: Activated Human T Cells Take Up Citrulline via L-Type Amino Acid Transporter 1 and Use It to Regenerate Arginine after Induction of Argininosuccinate Synthase Expression
Source: Front Immunol. 2017 Jul 24;8:864. doi: 10.3389/fimmu.2017.00864 (PMC5523021; doi:10.3389/fimmu.2017.00864)
Supplement: Supplementary file 1 [file Data_Sheet_1.pdf]

Supplementary Table 1:

The table lists all p-values between experimental groups obtained in the statistical analyses specified in the respective figure legend.

Figure 1

C

**experimental groups compared**	**p-value**
1000 µM Arg + Cit vs. 100 µM Arg + Cit	0,0017
1000 µM Arg + Cit vs. 20 µM Arg + Cit	< 0,0001
1000 µM Arg + Cit vs. 5 µM Arg + Cit	< 0,0001
1000 µM Arg + Cit vs. 0 µM Arg + Cit	< 0,0001
1000 µM Arg + Cit vs. 1000 µM Arg	0,3512
1000 µM Arg + Cit vs. 100 µM Arg	< 0,0001
1000 µM Arg + Cit vs. 20 µM Arg	< 0,0001
1000 µM Arg + Cit vs. 5 µM Arg	< 0,0001
1000 µM Arg + Cit vs. 0 µM Arg	< 0,0001
100 µM Arg + Cit vs. 20 µM Arg + Cit	< 0,0001
100 µM Arg + Cit vs. 5 µM Arg + Cit	< 0,0001
100 µM Arg + Cit vs. 0 µM Arg + Cit	< 0,0001
100 µM Arg + Cit vs. 1000 µM Arg	0,9142
100 µM Arg + Cit vs. 100 µM Arg	0,0065
100 µM Arg + Cit vs. 20 µM Arg	< 0,0001
100 µM Arg + Cit vs. 5 µM Arg	< 0,0001
100 µM Arg + Cit vs. 0 µM Arg	< 0,0001
20 µM Arg + Cit vs. 5 µM Arg + Cit	< 0,0001
20 µM Arg + Cit vs. 0 µM Arg + Cit	< 0,0001
20 µM Arg + Cit vs. 1000 µM Arg	< 0,0001
20 µM Arg + Cit vs. 100 µM Arg	0,2206
20 µM Arg + Cit vs. 20 µM Arg	< 0,0001
20 µM Arg + Cit vs. 5 µM Arg	< 0,0001
20 µM Arg + Cit vs. 0 µM Arg	< 0,0001
5 µM Arg + Cit vs. 0 µM Arg + Cit	0,7785
5 µM Arg + Cit vs. 1000 µM Arg	< 0,0001
5 µM Arg + Cit vs. 100 µM Arg	< 0,0001
5 µM Arg + Cit vs. 20 µM Arg	> 0,9999
5 µM Arg + Cit vs. 5 µM Arg	0,8368
5 µM Arg + Cit vs. 0 µM Arg	0,6058
0 µM Arg + Cit vs. 1000 µM Arg	< 0,0001
0 µM Arg + Cit vs. 100 µM Arg	< 0,0001
0 µM Arg + Cit vs. 20 µM Arg	0,4747
0 µM Arg + Cit vs. 5 µM Arg	> 0,9999
0 µM Arg + Cit vs. 0 µM Arg	> 0,9999
1000 µM Arg vs. 100 µM Arg	0,0003
1000 µM Arg vs. 20 µM Arg	< 0,0001
1000 µM Arg vs. 5 µM Arg	< 0,0001
1000 µM Arg vs. 0 µM Arg	< 0,0001

100 µM Arg vs. 20 µM Arg	< 0,0001
100 µM Arg vs. 5 µM Arg	< 0,0001
100 µM Arg vs. 0 µM Arg	< 0,0001
20 µM Arg vs. 5 µM Arg	0,5499
20 µM Arg vs. 0 µM Arg	0,3061
5 µM Arg vs. 0 µM Arg	> 0,9999

D

**experimental groups compared**	**p-value**
1000 µM Arg vs. 100 µM Arg	0,0060
1000 µM Arg vs. 20 µM Arg	< 0,0001
1000 µM Arg vs. 5 µM Arg	< 0,0001
1000 µM Arg vs. 0 µM Arg	< 0,0001
1000 µM Arg vs. 1000 µM Arg + Cit	0,1222
1000 µM Arg vs. 100 µM Arg + Cit	< 0,0001
1000 µM Arg vs. 20 µM Arg + Cit	< 0,0001
1000 µM Arg vs. 5 µM Arg + Cit	< 0,0001
1000 µM Arg vs. 0 µM Arg + Cit	< 0,0001
100 µM Arg vs. 20 µM Arg	0,0013
100 µM Arg vs. 5 µM Arg	< 0,0001
100 µM Arg vs. 0 µM Arg	< 0,0001
100 µM Arg vs. 1000 µM Arg + Cit	> 0,9999
100 µM Arg vs. 100 µM Arg + Cit	0,7467
100 µM Arg vs. 20 µM Arg + Cit	0,0054
100 µM Arg vs. 5 µM Arg + Cit	0,0001
100 µM Arg vs. 0 µM Arg + Cit	< 0,0001
20 µM Arg vs. 5 µM Arg	0,1931
20 µM Arg vs. 0 µM Arg	< 0,0001
20 µM Arg vs. 1000 µM Arg + Cit	0,0116
20 µM Arg vs. 100 µM Arg + Cit	0,2710
20 µM Arg vs. 20 µM Arg + Cit	> 0,9999
20 µM Arg vs. 5 µM Arg + Cit	0,9987
20 µM Arg vs. 0 µM Arg + Cit	0,0032
5 µM Arg vs. 0 µM Arg	0,4565
5 µM Arg vs. 1000 µM Arg + Cit	< 0,0001
5 µM Arg vs. 100 µM Arg + Cit	< 0,0001
5 µM Arg vs. 20 µM Arg + Cit	0,0750
5 µM Arg vs. 5 µM Arg + Cit	0,7580
5 µM Arg vs. 0 µM Arg + Cit	0,9393
0 µM Arg vs. 1000 µM Arg + Cit	< 0,0001
0 µM Arg vs. 100 µM Arg + Cit	< 0,0001
0 µM Arg vs. 20 µM Arg + Cit	< 0,0001
0 µM Arg vs. 5 µM Arg + Cit	0,0058
0 µM Arg vs. 0 µM Arg + Cit	0,9976

1000 µM Arg + Cit vs. 100 µM Arg + Cit	0,7267
1000 µM Arg + Cit vs. 20 µM Arg + Cit	0,0293
1000 µM Arg + Cit vs. 5 µM Arg + Cit	0,0019
1000 µM Arg + Cit vs. 0 µM Arg + Cit	< 0,0001
100 µM Arg + Cit vs. 20 µM Arg + Cit	0,5137
100 µM Arg + Cit vs. 5 µM Arg + Cit	0,0538
100 µM Arg + Cit vs. 0 µM Arg + Cit	< 0,0001
20 µM Arg + Cit vs. 5 µM Arg + Cit	0,9784
20 µM Arg + Cit vs. 0 µM Arg + Cit	0,0007
5 µM Arg + Cit vs. 0 µM Arg + Cit	0,0756

F

**experimental groups compared**	**p-value**
1 mM Arg vs. 0 mM Arg	< 0,0001
1 mM Arg vs. 0 mM Arg + 1 mM ASA	0,4226
0 mM Arg vs. 0 mM Arg + 1 mM ASA	< 0,0001

G

**experimental groups compared**	**p-value**
1 mM Arg vs. 0 mM Arg	< 0,0001
1 mM Arg vs. 0 mM Arg + 1 mM ASA	0,4641
0 mM Arg vs. 0 mM Arg + 1 mM ASA	< 0,0001

Figure 2

A

**experimental groups compared**	**p-value**
1000 µM Arg vs. 100 µM Arg	< 0,0001
1000 µM Arg vs. 20 µM Arg	< 0,0001
1000 µM Arg vs. 5 µM Arg	< 0,0001
1000 µM Arg vs. 0 µM Arg	< 0,0001
1000 µM Arg vs. 1000 µM Arg + Cit	0,9990
1000 µM Arg vs. 100 µM Arg + Cit	> 0,9999
1000 µM Arg vs. 20 µM Arg + Cit	< 0,0001
1000 µM Arg vs. 5 µM Arg + Cit	< 0,0001
1000 µM Arg vs. 0 µM Arg + Cit	< 0,0001
100 µM Arg vs. 20 µM Arg	< 0,0001
100 µM Arg vs. 5 µM Arg	< 0,0001
100 µM Arg vs. 0 µM Arg	< 0,0001
100 µM Arg vs. 1000 µM Arg + Cit	< 0,0001
100 µM Arg vs. 100 µM Arg + Cit	< 0,0001
100 µM Arg vs. 20 µM Arg + Cit	0,8939
100 µM Arg vs. 5 µM Arg + Cit	< 0,0001
100 µM Arg vs. 0 µM Arg + Cit	< 0,0001
20 µM Arg vs. 5 µM Arg	0,9981
20 µM Arg vs. 0 µM Arg	0,9977
20 µM Arg vs. 1000 µM Arg + Cit	< 0,0001
20 µM Arg vs. 100 µM Arg + Cit	< 0,0001
20 µM Arg vs. 20 µM Arg + Cit	0,0012
20 µM Arg vs. 5 µM Arg + Cit	> 0,9999
20 µM Arg vs. 0 µM Arg + Cit	0,9983
5 µM Arg vs. 0 µM Arg	> 0,9999
5 µM Arg vs. 1000 µM Arg + Cit	< 0,0001
5 µM Arg vs. 100 µM Arg + Cit	< 0,0001
5 µM Arg vs. 20 µM Arg + Cit	< 0,0001
5 µM Arg vs. 5 µM Arg + Cit	> 0,9999
5 µM Arg vs. 0 µM Arg + Cit	> 0,9999
0 µM Arg vs. 1000 µM Arg + Cit	< 0,0001
0 µM Arg vs. 100 µM Arg + Cit	< 0,0001
0 µM Arg vs. 20 µM Arg + Cit	< 0,0001
0 µM Arg vs. 5 µM Arg + Cit	0,9999
0 µM Arg vs. 0 µM Arg + Cit	> 0,9999
1000 µM Arg + Cit vs. 100 µM Arg + Cit	0,9983
1000 µM Arg + Cit vs. 20 µM Arg + Cit	< 0,0001
1000 µM Arg + Cit vs. 5 µM Arg + Cit	< 0,0001
1000 µM Arg + Cit vs. 0 µM Arg + Cit	< 0,0001
100 µM Arg + Cit vs. 20 µM Arg + Cit	< 0,0001
100 µM Arg + Cit vs. 5 µM Arg + Cit	< 0,0001
100 µM Arg + Cit vs. 0 µM Arg + Cit	< 0,0001
20 µM Arg + Cit vs. 5 µM Arg + Cit	0,0004
20 µM Arg + Cit vs. 0 µM Arg + Cit	< 0,0001

5 µM Arg + Cit vs. 0 µM Arg + Cit	> 0,9999

B

**experimental groups compared**	**p-value**
1000 µM Arg vs. 100 µM Arg	> 0,9999
1000 µM Arg vs. 20 µM Arg	< 0,0001
1000 µM Arg vs. 5 µM Arg	< 0,0001
1000 µM Arg vs. 0 µM Arg	< 0,0001
1000 µM Arg vs. 1000 µM Arg + Cit	0,9996
1000 µM Arg vs. 100 µM Arg + Cit	0,0464
1000 µM Arg vs. 20 µM Arg + Cit	< 0,0001
1000 µM Arg vs. 5 µM Arg + Cit	< 0,0001
1000 µM Arg vs. 0 µM Arg + Cit	< 0,0001
100 µM Arg vs. 20 µM Arg	< 0,0001
100 µM Arg vs. 5 µM Arg	< 0,0001
100 µM Arg vs. 0 µM Arg	< 0,0001
100 µM Arg vs. 1000 µM Arg + Cit	0,9328
100 µM Arg vs. 100 µM Arg + Cit	0,0020
100 µM Arg vs. 20 µM Arg + Cit	< 0,0001
100 µM Arg vs. 5 µM Arg + Cit	< 0,0001
100 µM Arg vs. 0 µM Arg + Cit	< 0,0001
20 µM Arg vs. 5 µM Arg	0,4075
20 µM Arg vs. 0 µM Arg	0,2630
20 µM Arg vs. 1000 µM Arg + Cit	< 0,0001
20 µM Arg vs. 100 µM Arg + Cit	< 0,0001
20 µM Arg vs. 20 µM Arg + Cit	< 0,0001
20 µM Arg vs. 5 µM Arg + Cit	> 0,9999
20 µM Arg vs. 0 µM Arg + Cit	0,3264
5 µM Arg vs. 0 µM Arg	> 0,9999
5 µM Arg vs. 1000 µM Arg + Cit	< 0,0001
5 µM Arg vs. 100 µM Arg + Cit	< 0,0001
5 µM Arg vs. 20 µM Arg + Cit	< 0,0001
5 µM Arg vs. 5 µM Arg + Cit	0,5764
5 µM Arg vs. 0 µM Arg + Cit	> 0,9999
0 µM Arg vs. 1000 µM Arg + Cit	< 0,0001
0 µM Arg vs. 100 µM Arg + Cit	< 0,0001
0 µM Arg vs. 20 µM Arg + Cit	< 0,0001
0 µM Arg vs. 5 µM Arg + Cit	0,4075
0 µM Arg vs. 0 µM Arg + Cit	> 0,9999
1000 µM Arg + Cit vs. 100 µM Arg + Cit	0,1229
1000 µM Arg + Cit vs. 20 µM Arg + Cit	< 0,0001
1000 µM Arg + Cit vs. 5 µM Arg + Cit	< 0,0001
1000 µM Arg + Cit vs. 0 µM Arg + Cit	< 0,0001
100 µM Arg + Cit vs. 20 µM Arg + Cit	< 0,0001
100 µM Arg + Cit vs. 5 µM Arg + Cit	< 0,0001
100 µM Arg + Cit vs. 0 µM Arg + Cit	< 0,0001

20 µM Arg + Cit vs. 5 µM Arg + Cit	< 0,0001
20 µM Arg + Cit vs. 0 µM Arg + Cit	< 0,0001
5 µM Arg + Cit vs. 0 µM Arg + Cit	0,4850

C

**experimental groups compared**	**p-value**
1000 µM Arg vs. 100 µM Arg	0,6202
1000 µM Arg vs. 20 µM Arg	< 0,0001
1000 µM Arg vs. 5 µM Arg	< 0,0001
1000 µM Arg vs. 0 µM Arg	< 0,0001
1000 µM Arg vs. 1000 µM Arg + Cit	0,0056
1000 µM Arg vs. 100 µM Arg + Cit	< 0,0001
1000 µM Arg vs. 20 µM Arg + Cit	< 0,0001
1000 µM Arg vs. 5 µM Arg + Cit	< 0,0001
1000 µM Arg vs. 0 µM Arg + Cit	< 0,0001
100 µM Arg vs. 20 µM Arg	< 0,0001
100 µM Arg vs. 5 µM Arg	< 0,0001
100 µM Arg vs. 0 µM Arg	< 0,0001
100 µM Arg vs. 1000 µM Arg + Cit	0,4940
100 µM Arg vs. 100 µM Arg + Cit	0,0002
100 µM Arg vs. 20 µM Arg + Cit	< 0,0001
100 µM Arg vs. 5 µM Arg + Cit	< 0,0001
100 µM Arg vs. 0 µM Arg + Cit	< 0,0001
20 µM Arg vs. 5 µM Arg	0,7202
20 µM Arg vs. 0 µM Arg	0,0440
20 µM Arg vs. 1000 µM Arg + Cit	< 0,0001
20 µM Arg vs. 100 µM Arg + Cit	0,1075
20 µM Arg vs. 20 µM Arg + Cit	> 0,9999
20 µM Arg vs. 5 µM Arg + Cit	0,7138
20 µM Arg vs. 0 µM Arg + Cit	0,0384
5 µM Arg vs. 0 µM Arg	0,9158
5 µM Arg vs. 1000 µM Arg + Cit	< 0,0001
5 µM Arg vs. 100 µM Arg + Cit	0,0002
5 µM Arg vs. 20 µM Arg + Cit	0,5405
5 µM Arg vs. 5 µM Arg + Cit	> 0,9999
5 µM Arg vs. 0 µM Arg + Cit	0,8994
0 µM Arg vs. 1000 µM Arg + Cit	< 0,0001
0 µM Arg vs. 100 µM Arg + Cit	< 0,0001
0 µM Arg vs. 20 µM Arg + Cit	0,0197
0 µM Arg vs. 5 µM Arg + Cit	0,9191
0 µM Arg vs. 0 µM Arg + Cit	> 0,9999
1000 µM Arg + Cit vs. 100 µM Arg + Cit	0,3861
1000 µM Arg + Cit vs. 20 µM Arg + Cit	< 0,0001
1000 µM Arg + Cit vs. 5 µM Arg + Cit	< 0,0001
1000 µM Arg + Cit vs. 0 µM Arg + Cit	< 0,0001
100 µM Arg + Cit vs. 20 µM Arg + Cit	0,1955

100 µM Arg + Cit vs. 5 µM Arg + Cit	0,0002
100 µM Arg + Cit vs. 0 µM Arg + Cit	< 0,0001
20 µM Arg + Cit vs. 5 µM Arg + Cit	0,5336
20 µM Arg + Cit vs. 0 µM Arg + Cit	0,0170
5 µM Arg + Cit vs. 0 µM Arg + Cit	0,9030

D

**experimental groups compared**	**p-value**
1000 µM Arg vs. 100 µM Arg	< 0,0001
1000 µM Arg vs. 20 µM Arg	< 0,0001
1000 µM Arg vs. 5 µM Arg	< 0,0001
1000 µM Arg vs. 0 µM Arg	< 0,0001
1000 µM Arg vs. 1000 µM Arg + Cit	< 0,0001
1000 µM Arg vs. 100 µM Arg + Cit	< 0,0001
1000 µM Arg vs. 20 µM Arg + Cit	< 0,0001
1000 µM Arg vs. 5 µM Arg + Cit	< 0,0001
1000 µM Arg vs. 0 µM Arg + Cit	< 0,0001
100 µM Arg vs. 20 µM Arg	< 0,0001
100 µM Arg vs. 5 µM Arg	< 0,0001
100 µM Arg vs. 0 µM Arg	< 0,0001
100 µM Arg vs. 1000 µM Arg + Cit	0,5805
100 µM Arg vs. 100 µM Arg + Cit	0,0593
100 µM Arg vs. 20 µM Arg + Cit	< 0,0001
100 µM Arg vs. 5 µM Arg + Cit	< 0,0001
100 µM Arg vs. 0 µM Arg + Cit	< 0,0001
20 µM Arg vs. 5 µM Arg	0,9843
20 µM Arg vs. 0 µM Arg	0,5765
20 µM Arg vs. 1000 µM Arg + Cit	< 0,0001
20 µM Arg vs. 100 µM Arg + Cit	0,0074
20 µM Arg vs. 20 µM Arg + Cit	0,9996
20 µM Arg vs. 5 µM Arg + Cit	0,9831
20 µM Arg vs. 0 µM Arg + Cit	0,6566
5 µM Arg vs. 0 µM Arg	0,9951
5 µM Arg vs. 1000 µM Arg + Cit	< 0,0001
5 µM Arg vs. 100 µM Arg + Cit	< 0,0001
5 µM Arg vs. 20 µM Arg + Cit	> 0,9999
5 µM Arg vs. 5 µM Arg + Cit	> 0,9999
5 µM Arg vs. 0 µM Arg + Cit	0,9982
0 µM Arg vs. 1000 µM Arg + Cit	< 0,0001
0 µM Arg vs. 100 µM Arg + Cit	< 0,0001
0 µM Arg vs. 20 µM Arg + Cit	0,9394
0 µM Arg vs. 5 µM Arg + Cit	0,9964
0 µM Arg vs. 0 µM Arg + Cit	> 0,9999
1000 µM Arg + Cit vs. 100 µM Arg + Cit	< 0,0001
1000 µM Arg + Cit vs. 20 µM Arg + Cit	< 0,0001
1000 µM Arg + Cit vs. 5 µM Arg + Cit	< 0,0001

1000 µM Arg + Cit vs. 0 µM Arg + Cit	< 0,0001
100 µM Arg + Cit vs. 20 µM Arg + Cit	0,0005
100 µM Arg + Cit vs. 5 µM Arg + Cit	0,0001
100 µM Arg + Cit vs. 0 µM Arg + Cit	< 0,0001
20 µM Arg + Cit vs. 5 µM Arg + Cit	> 0,9999
20 µM Arg + Cit vs. 0 µM Arg + Cit	0,9652
5 µM Arg + Cit vs. 0 µM Arg + Cit	0,9987

E

**experimental groups compared**	**p-value**
1 mM Arg vs. 0 mM Arg	< 0,0001
1 mM Arg vs. 1 mM ASA	0,0022
0 mM Arg vs. 1 mM ASA	< 0,0001

F

**experimental groups compared**	**p-value**
1 mM Arg vs. 0 mM Arg	< 0,0001
1 mM Arg vs. 1 mM ASA	0,0014
0 mM Arg vs. 1 mM ASA	< 0,0001

G

**experimental groups compared**	**p-value**
1 mM Arg vs. 0 mM Arg	< 0,0001
1 mM Arg vs. 1 mM ASA	< 0,0001
0 mM Arg vs. 1 mM ASA	< 0,0001

H

**experimental groups compared**	**p-value**
1 mM Arg vs. 0 mM Arg	< 0,0001
1 mM Arg vs. 1 mM ASA	< 0,0001
0 mM Arg vs. 1 mM ASA	0,0026

Figure 3

B

**experimental groups compared**	**p-value**
1000 µM Arg vs. 20 µM Arg	0,0504
1000 µM Arg vs. 0 µM Arg	< 0,0001
20 µM Arg vs. 0 µM Arg	< 0,0001

Figure 4

A

**experimental groups compared**	**p-value**
variation of time	0,0010
0 µM Arg vs. 20 µM Arg	0,4644

B

**experimental groups compared**	**p-value**
variation of time	0,0879
0 µM Arg vs. 20 µM Arg	0,0088

C

**experimental groups compared**	**p-value**
variation of time	0,6561
0 µM Arg vs. 20 µM Arg	0,0096

Figure 5

A

experimental groups compared	p-value
sodium (+) vs. sodium (-)	0,5243

B

experimental groups compared	p-value
control vs. Leu	< 0,0001
control vs. BCH	< 0,0001
control vs. His	< 0,0001
control vs. Arg	0,1101
control vs. Pro	0,1834
control vs. Gly	0,0105

Figure 6

A

**experimental groups compared**	**p-value**
control vs. Leu	< 0,0001
control vs. Cit	< 0,0001
Leu vs. Cit	< 0,0001

B

**experimental groups compared**	**p-value**
control vs. LAT1/4F2hc	< 0,0001

Figure 7

A

**experimental groups compared**	**p-value**
0 h:1000 µM Arg vs. 6 h:1000 µM Arg	< 0,0001
0 h:1000 µM Arg vs. 6 h:20 µM Arg	0,0001
0 h:1000 µM Arg vs. 6 h:0 µM Arg	< 0,0001
0 h:1000 µM Arg vs. 24 h:1000 µM Arg	0,0115
0 h:1000 µM Arg vs. 24 h:20 µM Arg	0,0004
0 h:1000 µM Arg vs. 24 h:0 µM Arg	0,0194
0 h:1000 µM Arg vs. 48 h:20 µM Arg	0,0001
0 h:1000 µM Arg vs. 48 h:0 µM Arg	0,0213
6 h:1000 µM Arg vs. 6 h:20 µM Arg	> 0,9999
6 h:1000 µM Arg vs. 6 h:0 µM Arg	0,8853
6 h:1000 µM Arg vs. 24 h:1000 µM Arg	0,9213
6 h:1000 µM Arg vs. 24 h:20 µM Arg	> 0,9999
6 h:1000 µM Arg vs. 24 h:0 µM Arg	0,8481
6 h:1000 µM Arg vs. 48 h:20 µM Arg	> 0,9999
6 h:1000 µM Arg vs. 48 h:0 µM Arg	0,8311
6 h:20 µM Arg vs. 6 h:0 µM Arg	0,9596
6 h:20 µM Arg vs. 24 h:1000 µM Arg	0,8984
6 h:20 µM Arg vs. 24 h:20 µM Arg	> 0,9999
6 h:20 µM Arg vs. 24 h:0 µM Arg	0,8206
6 h:20 µM Arg vs. 48 h:20 µM Arg	> 0,9999
6 h:20 µM Arg vs. 48 h:0 µM Arg	0,8032
6 h:0 µM Arg vs. 24 h:1000 µM Arg	0,0942
6 h:0 µM Arg vs. 24 h:20 µM Arg	0,6057
6 h:0 µM Arg vs. 24 h:0 µM Arg	0,0603
6 h:0 µM Arg vs. 48 h:20 µM Arg	0,8204
6 h:0 µM Arg vs. 48 h:0 µM Arg	0,0553
24 h:1000 µM Arg vs. 24 h:20 µM Arg	0,9954
24 h:1000 µM Arg vs. 24 h:0 µM Arg	> 0,9999
24 h:1000 µM Arg vs. 48 h:20 µM Arg	0,9580
24 h:1000 µM Arg vs. 48 h:0 µM Arg	> 0,9999
24 h:20 µM Arg vs. 24 h:0 µM Arg	0,9832
24 h:20 µM Arg vs. 48 h:20 µM Arg	> 0,9999
24 h:20 µM Arg vs. 48 h:0 µM Arg	0,9792
24 h:0 µM Arg vs. 48 h:20 µM Arg	0,9066
24 h:0 µM Arg vs. 48 h:0 µM Arg	> 0,9999
48 h:20 µM Arg vs. 48 h:0 µM Arg	0,8937

B

**experimental groups compared**	**p-value**
0 h:1000 µM Arg vs. 6 h:1000 µM Arg	0,4567
0 h:1000 µM Arg vs. 6 h:20 µM Arg	0,5617
0 h:1000 µM Arg vs. 6 h:0 µM Arg	0,0853
0 h:1000 µM Arg vs. 24 h:1000 µM Arg	> 0,9999

0 h:1000 µM Arg vs. 24 h:20 µM Arg	0,8946
0 h:1000 µM Arg vs. 24 h:0 µM Arg	0,0093
0 h:1000 µM Arg vs. 48 h:20 µM Arg	0,1151
0 h:1000 µM Arg vs. 48 h:0 µM Arg	0,0155
6 h:1000 µM Arg vs. 6 h:20 µM Arg	> 0,9999
6 h:1000 µM Arg vs. 6 h:0 µM Arg	0,9986
6 h:1000 µM Arg vs. 24 h:1000 µM Arg	0,8174
6 h:1000 µM Arg vs. 24 h:20 µM Arg	0,9998
6 h:1000 µM Arg vs. 24 h:0 µM Arg	0,8106
6 h:1000 µM Arg vs. 48 h:20 µM Arg	0,9997
6 h:1000 µM Arg vs. 48 h:0 µM Arg	0,8192
6 h:20 µM Arg vs. 6 h:0 µM Arg	0,9942
6 h:20 µM Arg vs. 24 h:1000 µM Arg	0,8909
6 h:20 µM Arg vs. 24 h:20 µM Arg	> 0,9999
6 h:20 µM Arg vs. 24 h:0 mM Arg	0,7177
6 h:20 µM Arg vs. 48 h:20 µM Arg	0,9982
6 h:20 µM Arg vs. 48 h:0 µM Arg	0,7351
6 h:0 µM Arg vs. 24 h:1000 µM Arg	0,2739
6 h:0 µM Arg vs. 24 h:20 µM Arg	0,8743
6 h:0 µM Arg vs. 24 h:0 µM Arg	0,9989
6 h:0 µM Arg vs. 48 h:20 µM Arg	> 0,9999
6 h:0 µM Arg vs. 48 h:0 mM Arg	0,9981
24 h:1000 µM Arg vs. 24 h:20 µM Arg	0,9958
24 h:1000 µM Arg vs. 24 h:0 µM Arg	0,0415
24 h:1000 µM Arg vs. 48 h:20 µM Arg	0,3441
24 h:1000 µM Arg vs. 48 h:0 µM Arg	0,0593
24 h:20 µM Arg vs. 24 h:0 µM Arg	0,3469
24 h:20 µM Arg vs. 48 h:20 µM Arg	0,9241
24 h:20 µM Arg vs. 48 h:0 mM Arg	0,3881
24 h:0 µM Arg vs. 48 h:20 µM Arg	0,9960
24 h:0 µM Arg vs. 48 h:0 µM Arg	> 0,9999
48 h:20 µM Arg vs. 48 h:0 µM Arg	0,9945

D

**experimental groups compared**	**p-value**
0 h:1000 µM Arg vs. 6 h:1000 µM Arg	0,9994
0 h:1000 µM Arg vs. 6 h:20 µM Arg	0,9998
0 h:1000 µM Arg vs. 6 h:0 µM Arg	> 0,9999
0 h:1000 µM Arg vs. 24 h:1000 µM Arg	< 0,0001
0 h:1000 µM Arg vs. 24 h:20 µM Arg	< 0,0001
0 h:1000 µM Arg vs. 24 h:0 µM Arg	0,3491
0 h:1000 µM Arg vs. 48 h:20 µM Arg	< 0,0001
0 h:1000 µM Arg vs. 48 h:0 µM Arg	0,0198
6 h:1000 µM Arg vs. 6 h:20 µM Arg	> 0,9999
6 h:1000 µM Arg vs. 6 h:0 µM Arg	> 0,9999
6 h:1000 µM Arg vs. 24 h:1000 µM Arg	0,0002

6 h:1000 µM Arg vs. 24 h:20 µM Arg	< 0,0001
6 h:1000 µM Arg vs. 24 h:0 µM Arg	0,8222
6 h:1000 µM Arg vs. 48 h:20 µM Arg	< 0,0001
6 h:1000 µM Arg vs. 48 h:0 µM Arg	0,1114
6 h:20 µM Arg vs. 6 h:0 µM Arg	> 0,9999
6 h:20 µM Arg vs. 24 h:1000 µM Arg	0,0002
6 h:20 µM Arg vs. 24 h:20 µM Arg	< 0,0001
6 h:20 µM Arg vs. 24 h:0 µM Arg	0,7672
6 h:20 µM Arg vs. 48 h:20 µM Arg	< 0,0001
6 h:20 µM Arg vs. 48 h:0 µM Arg	0,0904
6 h:0 µM Arg vs. 24 h:1000 µM Arg	< 0,0001
6 h:0 µM Arg vs. 24 h:20 µM Arg	< 0,0001
6 h:0 µM Arg vs. 24 h:0 µM Arg	0,5630
6 h:0 µM Arg vs. 48 h:20 µM Arg	< 0,0001
6 h:0 µM Arg vs. 48 h:0 µM Arg	0,0446
24 h:1000 µM Arg vs. 24 h:20 µM Arg	0,9988
24 h:1000 µM Arg vs. 24 h:0 µM Arg	0,0147
24 h:1000 µM Arg vs. 48 h:20 µM Arg	0,0034
24 h:1000 µM Arg vs. 48 h:0 µM Arg	0,2855
24 h:20 µM Arg vs. 24 h:0 µM Arg	0,0018
24 h:20 µM Arg vs. 48 h:20 µM Arg	0,0263
24 h:20 µM Arg vs. 48 h:0 µM Arg	0,0537
24 h:0 µM Arg vs. 48 h:20 µM Arg	< 0,0001
24 h:0 µM Arg vs. 48 h:0 µM Arg	0,9387
48 h:20 µM Arg vs. 48 h:0 µM Arg	< 0,0001

E

**experimental groups compared**	**p-value**
0 h:1000 µM Arg vs. 6 h:1000 µM Arg	0,7912
0 h:1000 µM Arg vs. 6 h:20 µM Arg	0,7983
0 h:1000 µM Arg vs. 6 h:0 µM Arg	0,9960
0 h:1000 µM Arg vs. 24 h:1000 µM Arg	< 0,0001
0 h:1000 µM Arg vs. 24 h:20 µM Arg	< 0,0001
0 h:1000 µM Arg vs. 24 h:0 µM Arg	0,0087
0 h:1000 µM Arg vs. 48 h:20 µM Arg	< 0,0001
0 h:1000 µM Arg vs. 48 h:0 µM Arg	< 0,0001
6 h:1000 µM Arg vs. 6 h:20 µM Arg	> 0,9999
6 h:1000 µM Arg vs. 6 h:0 µM Arg	0,9994
6 h:1000 µM Arg vs. 24 h:1000 µM Arg	0,0001
6 h:1000 µM Arg vs. 24 h:20 µM Arg	< 0,0001
6 h:1000 µM Arg vs. 24 h:0 µM Arg	0,3567
6 h:1000 µM Arg vs. 48 h:20 µM Arg	< 0,0001
6 h:1000 µM Arg vs. 48 h:0 µM Arg	0,0050
6 h:20 µM Arg vs. 6 h:0 µM Arg	0,9994
6 h:20 µM Arg vs. 24 h:1000 µM Arg	0,0001
6 h:20 µM Arg vs. 24 h:20 µM Arg	< 0,0001

6 h:20 µM Arg vs. 24 h:0 µM Arg	0,3497
6 h:20 µM Arg vs. 48 h:20 µM Arg	< 0,0001
6 h:20 µM Arg vs. 48 h:0 µM Arg	0,0049
6 h:0 µM Arg vs. 24 h:1000 µM Arg	< 0,0001
6 h:0 µM Arg vs. 24 h:20 µM Arg	< 0,0001
6 h:0 µM Arg vs. 24 h:0 µM Arg	0,0822
6 h:0 µM Arg vs. 48 h:20 µM Arg	< 0,0001
6 h:0 µM Arg vs. 48 h:0 µM Arg	0,0007
24 h:1000 µM Arg vs. 24 h:20 µM Arg	0,9965
24 h:1000 µM Arg vs. 24 h:0 µM Arg	0,0499
24 h:1000 µM Arg vs. 48 h:20 µM Arg	0,0001
24 h:1000 µM Arg vs. 48 h:0 µM Arg	0,9109
24 h:20 µM Arg vs. 24 h:0 µM Arg	0,0052
24 h:20 µM Arg vs. 48 h:20 µM Arg	0,0011
24 h:20 µM Arg vs. 48 h:0 µM Arg	0,3634
24 h:0 µM Arg vs. 48 h:20 µM Arg	< 0,0001
24 h:0 µM Arg vs. 48 h:0 µM Arg	0,6543
48 h:20 µM Arg vs. 48 h:0 µM Arg	< 0,0001

Figure 8

B

**experimental groups compared**	**p-value**
Co vs. -	0,0005
Co vs. nt	0,0003
Co vs. siLAT1	0,7887
- vs. nt	0,9951
- vs. siLAT1	0,0024
nt vs. siLAT1	0,0014

C

**experimental groups compared**	**p-value**
Co vs. -	< 0,0001
Co vs. nt	< 0,0001
Co vs. siLAT1	0,7093
- vs. nt	0,9701
- vs. siLAT1	< 0,0001
nt vs. siLAT1	< 0,0001
